# A Prophage-Encoded Small RNA Controls Metabolism and Cell Division in *Escherichia coli*

**DOI:** 10.1128/mSystems.00021-15

**Published:** 2016-02-09

**Authors:** Divya Balasubramanian, Preethi T. Ragunathan, Jingyi Fei, Carin K. Vanderpool

**Affiliations:** a Department of Microbiology, University of Illinois at Urbana–Champaign, Urbana, Illinois, USA; b Department of Microbiology, New York University School of Medicine, New York, New York, USA; c Department of Physics, Center for the Physics of Living Cells, University of Illinois at Urbana–Champaign, Urbana, Illinois, USA; d Department of Biochemistry and Molecular Biology, the University of Chicago, Chicago, Illinois, USA; University of California, Irvine

**Keywords:** DicB, DicB, FtsZ, FtsZ, Hfq, Hfq, RNase E, RNase E, cryptic prophage

## Abstract

Hundreds of small RNAs (sRNAs) have been identified in diverse bacterial species, and while the functions of most remain unknown, some regulate key processes, particularly stress responses. The sRNA DicF was identified over 25 years ago as an inhibitor of cell division but since then has remained uncharacterized. DicF consists of 53 nucleotides and is encoded by a gene carried on a prophage (Qin) in the genomes of many *Escherichia coli* strains*.* We demonstrated that DicF inhibits cell division via direct base pairing with *ftsZ* mRNA to repress translation and prevent new synthesis of the bacterial tubulin homolog FtsZ. Systems analysis using computational and experimental methods identified additional mRNA targets of DicF: *xylR* and *pykA* mRNAs, encoding the xylose uptake and catabolism regulator and pyruvate kinase, respectively. Genetic analyses showed that DicF directly base pairs with and represses translation of these targets. Phenotypes of cells expressing DicF variants demonstrated that DicF-associated growth inhibition is not solely due to repression of *ftsZ*, indicating that the physiological consequences of DicF-mediated regulation extend beyond effects on cell division caused by reduced FtsZ synthesis.

**IMPORTANCE** sRNAs are ubiquitous and versatile regulators of bacterial gene expression. A number of well-characterized examples in *E. coli* are highly conserved and present in the *E. coli* core genome. In contrast, the sRNA DicF (identified over 20 years ago but remaining poorly characterized) is encoded by a gene carried on a defective prophage element in many *E. coli* genomes. Here, we characterize DicF in order to better understand how horizontally acquired sRNA regulators impact bacterial gene expression and physiology. Our data confirm the long-hypothesized DicF-mediated regulation of *ftsZ*, encoding the bacterial tubulin homolog required for cell division. We further uncover DicF-mediated posttranscriptional control of metabolic gene expression. Ectopic production of DicF is highly toxic to *E. coli* cells, but the toxicity is not attributable to DicF regulation of *ftsZ.* Further work is needed to reveal the biological roles of and benefits for the host conferred by DicF and other products encoded by defective prophages.

## Abstract (precis)

sRNAs are ubiquitous and versatile regulators of bacterial gene expression. A number of well-characterized examples in *E. coli* are highly conserved and present in the *E. coli* core genome. In contrast, the sRNA DicF (identified over 20 years ago but remaining poorly characterized) is encoded by a gene carried on a defective prophage element in many *E. coli* genomes. Here, we characterize DicF in order to better understand how horizontally acquired sRNA regulators impact bacterial gene expression and physiology. Our data confirm the long-hypothesized DicF-mediated regulation of *ftsZ*, encoding the bacterial tubulin homolog required for cell division. We further uncover DicF-mediated posttranscriptional control of metabolic gene expression. Ectopic production of DicF is highly toxic to *E. coli* cells, but the toxicity is not attributable to DicF regulation of *ftsZ.* Further work is needed to reveal the biological roles of and benefits for the host conferred by DicF and other products encoded by defective prophages.

## INTRODUCTION

Many bacterial small RNAs (sRNAs) were fortuitously discovered starting in the 1980s, but it is only in the past decade that their roles as posttranscriptional regulators have been characterized in some detail. sRNA regulators that base pair with mRNA targets can exert positive or negative effects on mRNA translation or stability and typically require an RNA chaperone (Hfq) (1–3) to carry out their functions. Many bacterial sRNAs are produced in response to a specific stress (4–8), and sRNA-mediated regulation promotes adaptation to stress. Other sRNAs regulate housekeeping functions that help bacteria maintain metabolic homeostasis (9). The sRNA DicF was discovered in the 1980s, when it was observed that *dicF* in multicopy configuration inhibited cell division and caused filamentation (10, 11). The *dicF* gene is present in an operon encoding six gene products: DicF and five small proteins (12, 13). The promoter-proximal genes, *ydfA*, *ydfB*, and *ydfC*, encode small proteins of unknown function; these genes are followed by a long untranslated region containing *dicF* and then by the genes *dicB* and *ydfD* (13, 14). DicB, a 64-amino-acid protein, is the only other characterized gene product encoded by this operon (referred to historically and in this study as the *dicBF* operon). DicB is also a cell division inhibitor and carries out this function by (indirectly) preventing polymerization of FtsZ, the protein that forms the contractile ring for bacterial cell division (13, 15).

The 5′ end of DicF is produced by RNase E-mediated processing of the polycistronic transcript (13). Two different 3′ ends have been reported. Rho-independent transcription termination downstream of *ydfC* generates the 3′ end of a 53-nucleotide (nt) DicF sRNA. RNase III-mediated processing of the full-length *dicBF* mRNA yields a 3′ end that results in a 72-nt DicF molecule (13). DicF binds Hfq (16, 17) and was previously shown to inhibit *ftsZ* translation, an effect that was postulated to occur via an antisense base pairing mechanism (14). Consistent with this putative regulation, overproduction of DicF inhibited FtsZ protein synthesis and caused cell filamentation. Moreover, DicF overproduction led to aberrant nucleoid separation and to strong growth inhibition of *Escherichia coli* (14). Together, these observations indicate that overproduction of DicF is toxic to *E. coli*. However, the mechanistic basis of this toxicity has not been defined, and aside from *ftsZ*, no other putative targets of DicF have been identified.

DicF is encoded by a gene carried on Qin, a defective lambdoid prophage in the *E. coli* chromosome*.* Qin is one of nine *E. coli* K-12 defective prophages (18, 19) that have lost genes (by mutation or deletion) required for induction of the lytic life cycle, excision from the host chromosome, and/or production of progeny virions (20, 21). There are a number of well-characterized cases where genes carried by functional prophages are beneficial to the bacterial host (22–24). In contrast, the impacts of defective prophage genes on bacterial physiology have remained enigmatic. A recent study implicated *E. coli* defective prophages in modulation of many facets of cell growth and physiology, particularly in response to stresses (25). In that study, gene products encoded by Qin (particularly DicB) promoted resistance to the antibiotics azlocillin and nalidixic acid (25).

In this study, we probed the function of DicF by using global approaches to identify mRNA targets and by combining these with phenotypic comparison of cells expressing wild-type (wt) and mutant DicF RNAs. We found that, in addition to *ftsZ*, DicF also directly represses *xylR* and *pykA*, encoding the xylose regulator and pyruvate kinase, respectively*.* We demonstrated that the toxic effects of DicF are not solely attributable to regulation of *ftsZ*, suggesting that DicF regulation of other targets has important physiological consequences. Comparing the relative roles of DicF and DicB in growth inhibition of *E. coli* cells when the *dicBF* operon is ectopically expressed, we found that while both contribute, DicB is a more potent growth inhibitor than DicF.

## RESULTS

To identify putative targets of DicF, we used global computational and experimental approaches. Four computational programs, TargetRNA (26), IntaRNA (27), CopraRNA (28), and sTarPicker (29), which utilize different methods to predict base pairing interactions between sRNAs and potential target mRNAs (30), were used to generate a list of putative DicF targets. Targets predicted by at least two algorithms (with a *P* value of <0.03) are shown in Table S1 in the supplemental material. We also conducted transcriptome sequencing (RNA-Seq) analyses of an *E. coli* Δ*dicF* mutant harboring vector or P_lac_-*dicF* plasmids. RNA-Seq data were analyzed from 3 independent biological replicates and differentially expressed genes identified as described previously (31) (see Table S2). Candidates prioritized for further study had ≥100 normalized read counts (reads per kilobase per million [RPKM] values) under at least one set of experimental conditions and were 3-fold upregulated or downregulated in their levels compared to a vector control (*q* value of ≤0.05) (see Table S2).

10.1128/mSystems.00021-15.6 Table S1  Predicted interactions between DicF and targets. Regions of base pairing interactions between selected target mRNAs and DicF are indicated. Download Table S1, DOCX file, 0.02 MB. Copyright © 2016 Balasubramanian et al. 2016 Balasubramanian et al. This content is distributed under the terms of the Creative Commons Attribution 4.0 International license.

10.1128/mSystems.00021-15.7 Table S2  RNA-Seq results of differential gene expression upon ectopic expression of DicF. Data were analyzed as described in Materials and Methods. Normalized read counts are given for *E. coli* strains carrying a vector control (vector) or plasmid expressing *dicF* (DicF). Download Table S2, XLSX file, 0.4 MB. Copyright © 2016 Balasubramanian et al. 2016 Balasubramanian et al. This content is distributed under the terms of the Creative Commons Attribution 4.0 International license.

### Validation of posttranscriptional regulation by DicF.

**FIG 1:**
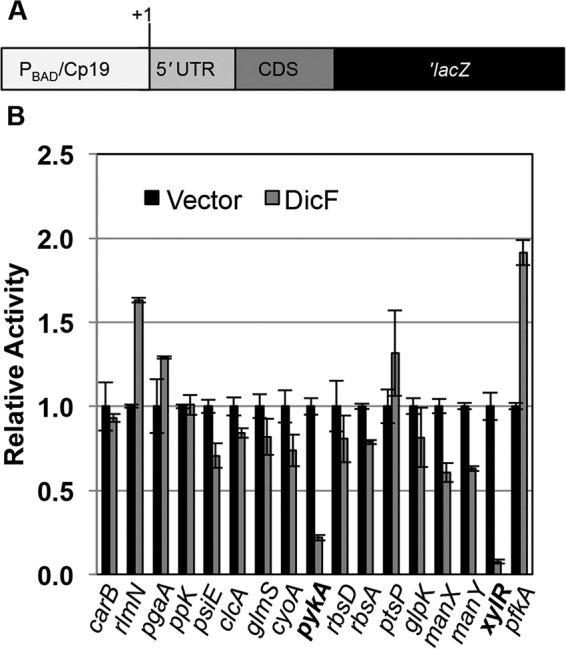
The genes *pykA* and *xylR* are posttranscriptionally regulated by DicF. (A) A schematic of reporter fusion constructs used in this experiment. Selected candidates from Table 1 were fused to ′*lacZ* and integrated as single copies on the *E. coli* chromosome, under the control of either an arabinose-inducible (P_BAD_) promoter or a constitutive (Cp19) promoter (see text), followed by the 5′ untranslated region (5′ UTR) of the gene and 10 to 20 amino acids of the coding sequence. (B) The fusions created in a wt *E. coli* host strain were assayed for β-galactosidase activity after DicF expression from a plasmid. The specific activities (quantified in Miller units) were normalized to the vector control of the corresponding strain to yield relative-activity data. The targets chosen for further study (indicated in bold) were up- or downregulated by DicF at least 2-fold.

Chromosomal translational *lacZ* fusions were constructed for a subset of candidates (Table 1). Fragments encompassing the 5′ untranslated region (UTR) and 10 to 20 amino acids of coding sequence (CDS) of each target were placed under the control of an inducible promoter (P_BAD_ [32]) or a constitutive promoter (Cp19 [33]) (Fig. 1A) to eliminate indirect effects of DicF on target gene transcription. Wild-type *E. coli* reporter strains carrying either the vector control or a *dicF* plasmid were induced with IPTG (isopropyl-β-d-thiogalactopyranoside), and β-galactosidase activity was monitored. The activity of several fusions was slightly altered by ectopic production of DicF (Fig. 1B), but only *xylR* and *pykA* fusions exhibited a ≥2-fold difference in activity in response to DicF. RNA-Seq showed that levels of *ftsZ* mRNA were reduced by ~1.7-fold in DicF-expressing cells (see Table S2 in the supplemental material), and since previous studies suggested *ftsZ* mRNA as a DicF target, we included it in downstream analyses.

**TABLE 1:** Target candidates for DicF sRNA^a^

Category	Annotation	Gene(s)	Fold change^b^ (pDicF/vec)	*q* value	Comp. predicted
Transport of sugars	Maltose transport, metabolism	*malM*, *malF*, *malG*, *malP*, *malE*^c^	0.003–0.01	0	
	Ribose transport, metabolism	*rbsD*	0.02	0	
		*rbsA*	0.03	0	
		*rbsC*	0.04	0	x
		*rbsB*	0.05	0	
		*rbsK*	0.1	0	
		*rbsR*	0.15	2.9E^−186^	
	Mannose and glucose transport	*manX*	0.31	1.0E^−134^	x
		*manY*	0.23	4.4E^−122^	
		*manZ*	0.24	2.1E^−153^	
	Galactitol transport and metabolism	*gatB*	0.03	0	
		*gatC*, *gatD*^c^	0.04	0	
		*gatZ*	0.05	0	
		*gatY*, *gatA*^c^	0.06	0	
	Xylose transport operon activator	*xylR^d^*	0.67	1	x
Central/carbon metabolism	Pyruvate kinase	*pykA*	0.1	0	x
	Glycerol kinase	*glpK*	0.06	0	x
	Succinate dehydrogenase	*sdhA*, *sdhB*, *sdhC*^c^	0.08–0.1	0	
	NADH:ubiquinone oxidoreductase	*nuoF*	0.2	0	
		*nuoE*	0.2	3.46E^−163^	
		*nuoC*	0.24	7.82E^−123^	
		*nuoG*	0.25	9.1E^−201^	
		*nuoB*	0.25	7.6E^−123^	
		*nuoL*	0.29	6.17E^−98^	x
		*nuoA*	0.27	6.72E^−98^	
	Cytochrome oxidase subunit II	*cyoA^e^*	0.5	1.63E^−22^	
	l-Glutamine:d-fructose-6-phosphate aminotransferase	*glmS^f^*	0.7	6.6E^−4^	
	PTS enzyme	*ptsP^d^*	1.02	0.11	x
	Phosphofructokinase	*pfkA^f^*	1.6	5.57E^−11^	
Miscellaneous	Tubulin-like cell division protein	*ftsZ^g^*	0.6	4.3E^−12^	x
	Polyphosphate kinase	*ppK^d^*	0.9	0.44	x
	Methyltransferase for rRNA	*rlmN^g^*	1.6	1.2E^−8^	x
	Tryptophan transporter	*mtr^d^*	1.5	1	x
	Carbamyl phosphatase synthase	*carB^d^*	2.13	1	x
	Polysaccharide production	*pgaA^d^*	2.5	1	x
	Phosphate starvation	*psiE^d^*	8	1	x

a RNA-Seq experiments and biocomputational analyses were used to generate a list of potential DicF targets. Each “x” in the “Comp. predicted” column indicates that the results were predicted by a computational program. PTS, phosphotransferase system.

b Data represent normalized read counts from cells carrying P*_lac_*-*dicF* plasmid divided by counts from vector control (vec) cells. Values of <1 indicate repression by DicF; values of >1 indicate activation by DicF.

c The fold change and *q* values for the gene were the same.

d The gene did not meet the normalized read count cutoff (it had <100 reads) but was chosen because it were predicted by computational programs, as described in the text.

e The gene did not meet the cutoff fold change for RNA-Seq analyses but was chosen because it was 2-fold downregulated and because several genes encoding other components of the enzyme were also downregulated ~2-fold.

f The gene did not meet the cutoff fold change and *q* values for RNA-Seq data analyses (although it fulfilled the copy number criteria) but was chosen because it was predicted by computational programs, as described in the text.

g The gene neither fulfilled the RNA-Seq cutoff fold change criteria nor was predicted by computational programs but was chosen based on other experimental evidence.

### Posttranscriptional regulation of targets by DicF does not require other Qin functions.

**FIG 2:**
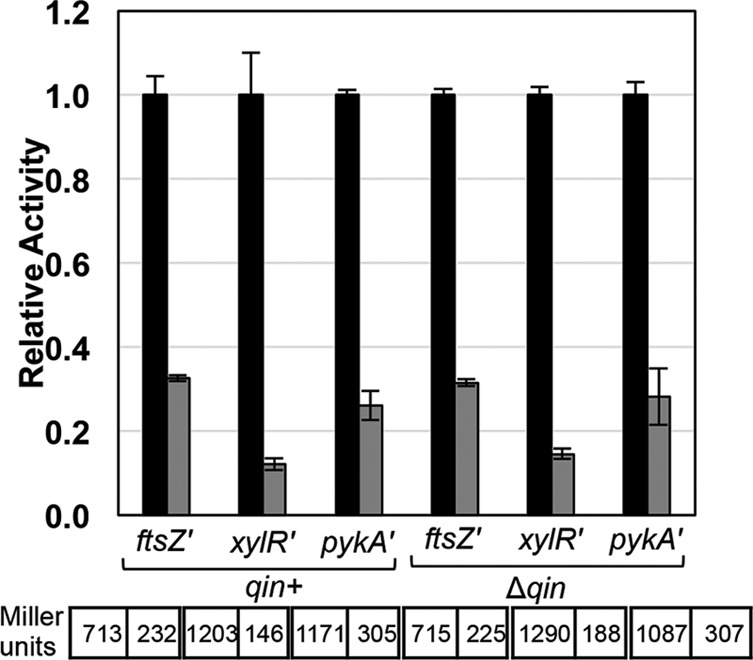
Regulation of *ftsZ*, *xylR*, and *pykA* by DicF does not require other factors carried on Qin prophage. β-Galactosidase activity of *ftsZ*′*-*′*lacZ*, *xylR*′*-*′*lacZ*, and *pykA*′*-*′*lacZ* after DicF expression was assayed in the indicated strain backgrounds*.* The specific activities in Miller units (indicated at the bottom) were normalized to the corresponding vector control strains to yield relative-activity data for the experimental strain.

Several genes carried on Qin were differentially regulated upon DicF production (see Table S2 in the supplemental material). To determine whether *xylR*, *pykA*, and *ftsZ* regulation by DicF required other Qin-encoded functions, we examined DicF regulation of these targets in a host strain where the entire *qin* prophage (~20 kb) was deleted (constructed as described in reference 25). As shown in Fig. 2, *xylR*, *pykA*, and *ftsZ* were regulated by DicF in the presence and absence of *qin*, indicating that regulation of these targets by DicF is not mediated indirectly by other Qin-encoded functions.

### Characterization of DicF regulation of *ftsZ* mRNA.

**FIG 3:**
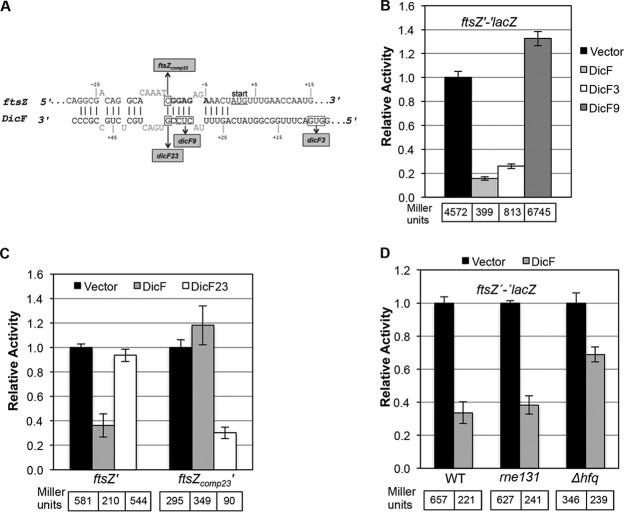
Genetic and molecular characterization of DicF-*ftsZ* mRNA interactions. (A) Base pairing predictions for *ftsZ* mRNA and DicF. The numbers for *ftsZ* are relative to the start codon (with A as +1). The numbers for DicF are relative to the 5′ end of *dicF.* The predicted *ftsZ* RBS is shown in bold, and the start codon is underlined. The predicted base pairing interactions between *ftsZ* and DicF are marked by vertical lines, and mutations in *dicF* and *ftsZ* are indicated by boxes. All mutations were substitutions, and boxed residues were changed to the complementary nucleotides. Nonpaired nucleotides or gaps are indicated by sequences above or below paired nucleotides. (B) The β-galactosidase activities of the *ftsZ*′*-*′*lacZ* fusion strains ectopically producing DicF, DicF3, or DicF9 were assayed. (C) The β-galactosidase activities of the *ftsZ*′*-*′*lacZ* fusion and the compensatory *ftsZ_comp23_*′*-*′*lacZ* fusion were assayed upon overexpression of DicF and DicF23. (D) The levels of β-galactosidase activity of the *ftsZ*′*-*′*lacZ* fusion in the wild-type (WT) strain and in the *rne131* and *hfq* mutants were assayed after overexpression of DicF. The specific activities of the fusions were normalized as described for Fig. 2.

The 3′ region of DicF is predicted to base pair with the *ftsZ* mRNA ribosome binding site (RBS) (14) (Fig. 3A). To test this prediction, we examined regulation of an *ftsZ*′*-*′*lacZ* translational fusion by wt DicF, DicF9, and DicF3 (Fig. 3A). The DicF9 mutation disrupts the predicted DicF-*ftsZ* mRNA interaction, while the DicF3 mutation does not. Wild-type DicF and DicF3 strongly repressed *ftsZ* translation, while DicF9 failed to regulate *ftsZ* (Fig. 3B). To further confirm direct interactions between DicF and the *ftsZ* mRNA, we constructed DicF23 and *ftsZ_comp23_*′*-*′*lacZ* (*ftsZ_comp23_* contains compensatory mutations to restore pairing with DicF23) mutations to disrupt and restore the base pairing interaction upstream of the *ftsZ* RBS (Fig. 3A). DicF23 did not repress wt *ftsZ* translation as efficiently as wt DicF; likewise, wt DicF did not efficiently repress *ftsZ_comp23_* (Fig. 3C). Importantly, DicF23 regulated *ftsZ_comp23_* at nearly wt levels (Fig. 3C). These data are consistent with the long-held hypothesis (14) that DicF directly represses the essential *E. coli* gene *ftsZ* via direct base pairing interactions.

Many sRNAs require Hfq (34–36) for their stability and for interactions with targets (e.g., SgrS [37, 38], RydC [39], and OxyS/RprA [40]), and the RNase E degradosome is important for degradation of sRNA-mRNA complexes in the context of negative regulation (41–43). To assess whether Hfq and the degradosome are necessary for regulation of *ftsZ* by DicF, we tested the activity of the *ftsZ*′*-*′*lacZ* fusion in wt, *hfq*, and *rne131* (degradosome mutant) backgrounds. Hfq was important for DicF regulation of *ftsZ*, as repression in the *hfq* mutant was less stringent than that seen with the wt strain (Fig. 3D). However, *ftsZ* translation was still efficiently repressed by DicF in the *rne131* background, suggesting that DicF-mediated translational silencing of *ftsZ* does not absolutely require RNase E-mediated degradation. Nonetheless, in the context of the full-length *ftsZ* mRNA, translational repression and mRNA degradation may indeed be coupled.

### Characterization of DicF regulation of *xylR* mRNA.

**FIG 4:**
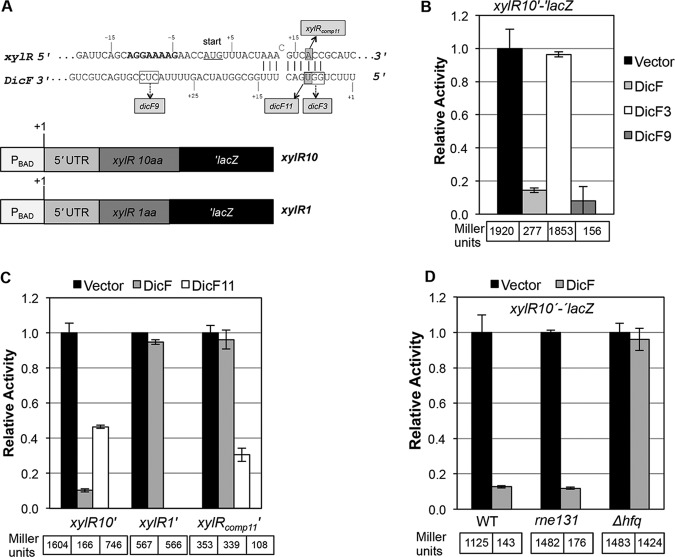
Genetic and molecular characterization of DicF-*xylR* mRNA interactions. (A) Base pairing predictions for *xylR* mRNA and DicF. The numbers for *xylR* are relative to the start codon (with A as +1). The numbers for DicF are relative to the 5′ end of *dicF.* The *xylR* RBS is shown in bold, and the start codon is underlined. The predicted base pairing interactions between *xylR* and DicF are marked by vertical lines, and mutations in *dicF* and *xylR* are indicated by boxes. All mutations were substitutions, and the boxed residues were changed to the complementary nucleotides. The cartoon depicts the *xylR*′*-*′*lacZ* fusion containing 10 codons or 1 codon of the *xylR* gene (*xylR10* or *xylR1*, respectively). (B) The β-galactosidase activities of the *xylR10*′*-*′*lacZ* fusions were assayed during overexpression of DicF, DicF3, and DicF9. (C) The *xylR10*, *xylR1*, and *xylR_comp11_*′-′*lacZ* fusions were assayed for β-galactosidase activity during DicF and DicF11 overproduction. (D) The levels of β-galactosidase activity of the *xylR10*′-′*lacZ* fusion in the wt strain and the *rne131* and *hfq* mutants were assayed during overexpression of DicF. The specific activities of the fusions were normalized as described for Fig. 2.

XylR is a transcription factor that activates d-xylose import (*xylFGH*) and catabolism (*xylAB*) genes. A *xylR* translational fusion was strongly repressed by DicF (Fig. 1). While sequences near the 3′ end of DicF are involved in interactions with *ftsZ* mRNA, the 5′ end of DicF was predicted to interact with *xylR* mRNA (Fig. 4A)*.* DicF3 and DicF9 were tested alongside wt DicF for regulation of *xylR10*′*-*′*lacZ* (Fig. 4B). In contrast with the result for *ftsZ* (Fig. 3B), DicF9 still strongly regulated *xylR* translation, while DicF3 failed to regulate *xylR* (Fig. 4B). These results suggest that DicF interacts with *xylR* and *ftsZ* mRNAs using distinct residues in the 5′ and 3′ ends, respectively.

To further characterize DicF regulation of *xylR* and verify the predicted interaction (Fig. 4A), a translational fusion to the *xylR* start codon (lacking the predicted DicF binding site) was constructed (*xylR1*′*-*′*lacZ*; Fig. 4A). As predicted, wt DicF failed to regulate the truncated fusion (Fig. 4C). Point mutations in *dicF* (DicF11) and *xylR* (*xylR_comp11_*) that disrupted and then restored complementarity confirmed the regulation (Fig. 4C). The mutation in DicF11 relieved regulation of wt *xylR*, and the *xylR_comp11_* mutation likewise prevented regulation by wt DicF. Regulation was restored for the DicF11-*xylR_comp11_* compensatory pair (Fig. 4C). DicF-mediated translational regulation of the *xylR* reporter fusion did not require the RNase E degradosome but did require Hfq (Fig. 4D). There was little change in *xylR* mRNA levels in response to DicF (see Fig. S1A in the supplemental material), suggesting that the primary mechanism of *xylR* regulation is translational repression and not mRNA decay.

10.1128/mSystems.00021-15.1 Figure S1  Effects of DicF on *xylR* and *pykA* mRNA levels. A Cp19-*xylR pykA lacI*^q^ (DB223 and DB224) strain harboring the P_lac_-vector or P_lac_-*dicF* was grown to an OD_600_ of 0.1, 0.5 mM IPTG was added, and total RNA was extracted at the time points indicated. RNA was run on an agarose gel with a ladder (lanes M) and subjected to Northern blotting and probing for *xylR* (A) and *pykA* (B) using the corresponding biotinylated probes. A biotinylated *ssrA* probe was used as the loading control. Download Figure S1, PDF file, 0.3 MB. Copyright © 2016 Balasubramanian et al. 2016 Balasubramanian et al. This content is distributed under the terms of the Creative Commons Attribution 4.0 International license.

**FIG 5:**
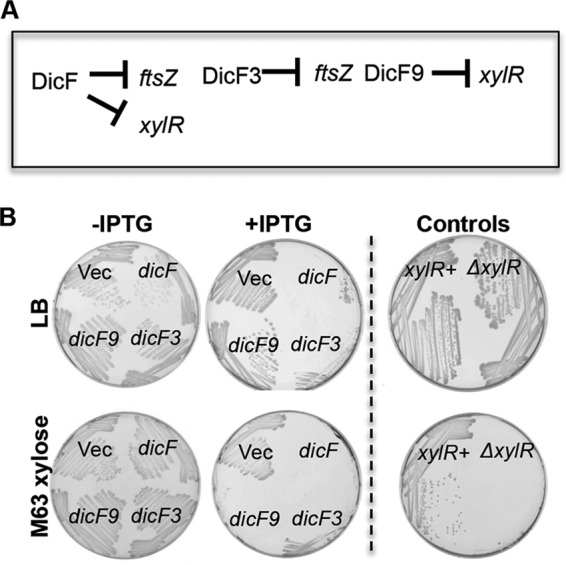
Physiological consequences of DicF-*xylR* mRNA interactions. (A) Schematic depicting regulatory interactions between DicF and *xylR*. The flattened arrowheads indicate translational repression of *xylR* and *ftsZ* by the indicated DicF RNAs. (B) Δ*dicF lacI*^q^ strains harboring the P_lac_-vector or P_lac_-*dicF*, P_lac_-*dicF3*, or P_lac_-*dicF9* were streaked on LB agar plates or M63 xylose plates with and without 0.5 mM IPTG. LB plates were incubated at 37°C for 12 h and M63 xylose plates for 22 h.

Since XylR is a transcription factor that activates expression of xylose uptake and catabolism genes (44), repression of *xylR* by DicF should limit growth of *E. coli* in minimal medium with xylose as the sole carbon source. Cells expressing wt DicF were severely growth inhibited (as observed previously [14, 45]) on both LB and xylose minimal medium (Fig. 5; +IPTG, *dicF*). To differentiate the ability of DicF to restrict growth of *E. coli* specifically on xylose medium due to repression of *xylR* from general restriction of colony-forming ability due to repression of *ftsZ*, we utilized the *dicF3* and *dicF9* mutants (Fig. 3A and 4A), which differentially regulate *ftsZ* and *xylR*, respectively (Fig. 5). Cells producing DicF3 were growth inhibited on both LB and xylose media, as expected because DicF3 represses *ftsZ* (Fig. 3B). DicF9, which does not repress *ftsZ* (Fig. 3B) but which still represses *xylR* (Fig. 4B), allowed growth on LB (Fig. 5) as well as on minimal glucose and fructose plates (see Fig. S2 in the supplemental material) but inhibited growth on minimal xylose plates (Fig. 5). The control *xylR* mutant strain grew well on LB but was unable to grow on xylose minimal medium (Fig. 5). These growth phenotypes are entirely consistent with genetic data indicating that DicF uses different residues to base pair with different targets, *ftsZ* and *xylR.* Further, the data indicate that independent regulation of these two different targets results in specific phenotypes consistent with known physiological roles of the mRNA targets.

10.1128/mSystems.00021-15.2 Figure S2  Both DicF and DicF3 inhibit growth of *E. coli* in minimal medium containing glucose or fructose. Δ*dicF lacI*^q^ strains harboring the P_lac_-vector or P_lac_-*dicF*, -*dicF3*, or -*dicF9* were streaked on LB agar, M63 glucose, or fructose plates with and without 0.5 mM IPTG. LB plates were incubated at 37°C for 12 h and M63 glucose and fructose plates for 20 h. Download Figure S2, PDF file, 0.6 MB. Copyright © 2016 Balasubramanian et al. 2016 Balasubramanian et al. This content is distributed under the terms of the Creative Commons Attribution 4.0 International license.

### Characterization of DicF regulation of *pykA* mRNA.

**FIG 6:**
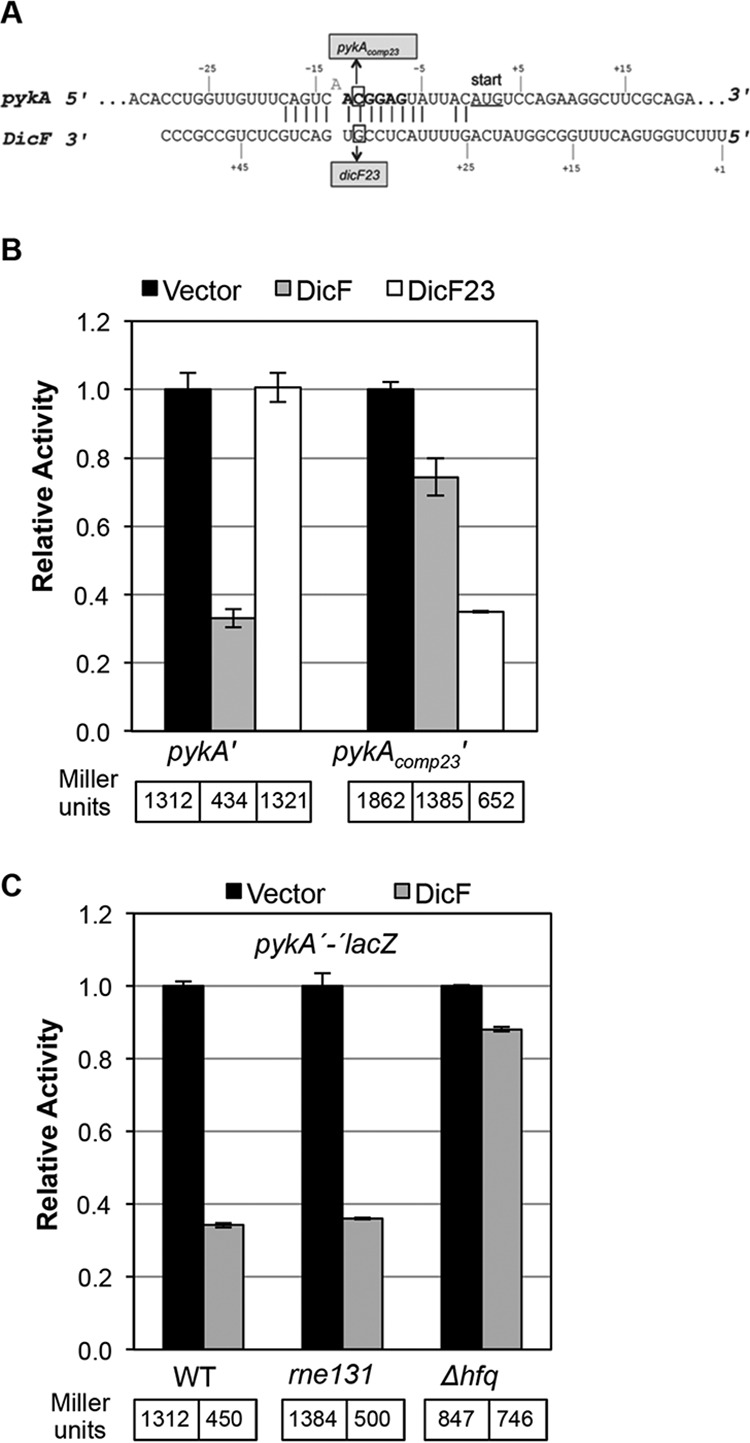
Genetic and molecular characterization of DicF-*pykA* mRNA interactions. (A) Base pairing predictions for *pykA* mRNA and DicF. The numbers for *pykA* are relative to the start codon (with A as +1). The numbers for DicF are relative to the 5′ end of *dicF.* The *pykA* RBS is shown in bold, and the start codon is underlined. The predicted base pairing interactions between *pykA* and DicF are marked by vertical lines, and mutations in *dicF* and *pykA* are indicated by boxes. All mutations were substitutions, and boxed residues were changed to the complementary nucleotides. (B) The levels of β-galactosidase activities of the *pykA* translational fusion were assayed during overexpression of DicF and DicF23. (C) The levels of β-galactosidase activity of the *pykA*′*-*′*lacZ* fusion in the wt strain and the *rne131* and *hfq* mutants were assayed during overexpression of DicF. The specific activities of the fusions were normalized as described for Fig. 2.

In *E. coli*, *pykA* encodes one of two pyruvate kinase isozymes that catalyze the conversion of phosphoenolpyruvate and ADP to pyruvate and ATP. DicF is predicted to interact with sequences encompassing the *pykA* mRNA RBS (Fig. 6A). The point mutation in DicF23 completely abolished repression of wt *pykA*′*-*′*lacZ* (Fig. 6A and B). A compensatory mutation in *pykA* (*pykA_comp23_*) impaired regulation by wt DicF but restored regulation by DicF23 (Fig. 6B)*.* These results validated the base pairing prediction and demonstrated that DicF directly regulates *pykA*. As for the other two targets, DicF regulation of *pykA* reporter translation requires Hfq but is not dependent on the presence of a functional degradosome (Fig. 6C). However, DicF was responsible for reduced levels of the *pykA* native transcript, as observed by Northern blot analysis (see Fig. S1B in the supplemental material), consistent with RNA-Seq analyses showing that *pykA* mRNA levels were strongly diminished upon DicF expression (Table 1). Together, these results suggest that, while translational regulation of *pykA* by DicF does not require the degradosome, the degradation of *pykA* mRNA may nevertheless play a role in regulation *in vivo*.

### Effects of DicF on *E. coli* growth and cell division.

**FIG 7:**
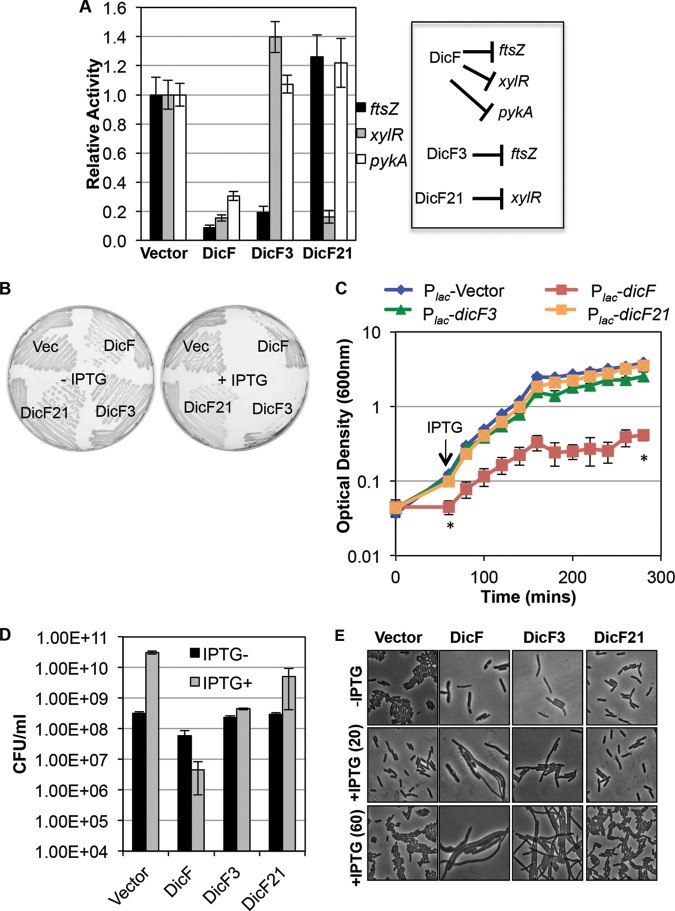
DicF inhibits cell division and prevents biomass increases. (A) The strains with *xylR*′, *pykA*′, and *ftsZ*′*-*′*lacZ* fusions and plasmids containing *dicF*, *dicF3*, or *dicF21* were assayed for β-galactosidase activity, after induction of the *dicF* alleles. A schematic representing regulation of the various targets by DicF and the mutants is on the right. (B) Δ*dicF lacI*^q^ strains carrying either the vector control or the *dicF* alleles shown in the figure were streaked on LB plates with and without 0.5 mM IPTG. (C) Δ*dicF* lacI^q^ strains carrying either the vector control or various *dicF* alleles was grown, 0.5 mM IPTG was added to the cultures, and growth was monitored over time. (D) The viability of the strains described for panel C was measured at time points indicated by asterisks. (E) Phase-contrast images of strains described for panel C imaged before (-IPTG) and 20 min [+IPTG (20)] and 60 min [+IPTG (60)] after addition of 0.5 mM IPTG.

A large number of genes were differentially expressed in control cells versus DicF-expressing cells (Table 1; see also Table S2 in the supplemental material). Our results thus far demonstrate that DicF directly regulates, at a minimum, three different mRNA targets in *E. coli*. While characterizing DicF regulation of *ftsZ*, *xylR*, and *pykA*, we isolated mutants that differentially regulate these targets (e.g., DicF3 and DicF9; Fig. 3, 4, and 5). Mutant DicF RNAs were produced at levels comparable to those seen with wt DicF, confirming that the mutations did not substantially alter DicF stability (see Fig. S3). We therefore utilized *dicF* mutants to further probe DicF-associated phenotypes. DicF3 represses *ftsZ* but fails to repress *xylR* and *pykA*, whereas DicF21 (see Fig. S4) does not regulate *ftsZ* or *pykA* but retains the ability to silence *xylR* translation (Fig. 7A). Growing on LB plates, *E. coli* strains expressing wt *dicF* or *dicF3* were unable to form individual colonies (Fig. 7B; +IPTG). In contrast, cells carrying the vector and cells expressing *dicF21* grew similarly on LB plates (Fig. 7B; +IPTG). Strains expressing the same alleles were cultured in liquid LB medium without inducer until mid-log phase, and then *dicF* expression was induced and growth (Fig. 7C) and viability (Fig. 7D) were monitored. Compared to control cells, DicF-expressing cells were severely growth inhibited (Fig. 7C) and viability was reduced by ~10-fold (Fig. 7D). In contrast, DicF3 cells continued to increase in optical density (OD), and CFU counts remained stable over the course of the experiment (Fig. 7C and D). (We note that the phenotype for DicF3-producing cells seems less severe than would be expected based on growth on LB plates [Fig. 7B]. In liquid media, cells were allowed to grow without induction for several generations and then *dicF* alleles were induced for ~3 h. Cells growing on plates with IPTG expressed *dicF* immediately and constitutively upon subculture to the plates, which might exacerbate the growth inhibition phenotypes.) Both wt DicF and DicF3 repressed *ftsZ*, as evidenced by filamentation of cells expressing wt DicF or DicF3 (Fig. 7E). In contrast, cells producing DicF21, which regulates *xylR* but not *ftsZ* or *pykA*, showed growth (Fig. 7C), viability (Fig. 7D), and morphology (Fig. 7E) similar to those seen with control cells. Since wt DicF and DicF3 both repress *ftsZ* and inhibit cell division, but only wt DicF strongly inhibits growth of *E. coli* in liquid medium, we infer that DicF-dependent regulation of targets other than *ftsZ* (disrupted by the mutation in DicF3) substantially contributes to growth inhibition of cells by wt DicF. In other words, while DicF repression of *ftsZ* certainly inhibits cell division, this regulatory interaction does not account for the toxicity of DicF in *E. coli* cells.

10.1128/mSystems.00021-15.3 Figure S3  Levels of DicF from *dicF* alleles used in this study. Δ*dicF lacI*^q^ strains harboring the P_lac_-vector or P_lac_-*dicF* alleles used in this study were grown to an OD_600_ of 0.1, 0.5 mM IPTG was added, and RNA was extracted from cells at the indicated time points. Equal amounts of total RNA were resolved on a polyacrylamide gel and subjected to Northern blotting. The blot was then probed for DicF and the loading control (*ssrA*). Download Figure S3, PDF file, 0.2 MB. Copyright © 2016 Balasubramanian et al. 2016 Balasubramanian et al. This content is distributed under the terms of the Creative Commons Attribution 4.0 International license.

10.1128/mSystems.00021-15.4 Figure S4  Illustration of the *dicF21* allele. The *dicF21* allele used as described for Fig. 7 is indicated in bold (CAU). These sequences were mutated to GUA. Predicted base pairing regions of *dicF* corresponding to *xylR*, *pykA*, and *ftsZ* are indicated by asterisks. Download Figure S4, PDF file, 0.02 MB. Copyright © 2016 Balasubramanian et al. 2016 Balasubramanian et al. This content is distributed under the terms of the Creative Commons Attribution 4.0 International license.

Microscopy revealed subtle differences in morphologies between cells expressing wt DicF and those expressing DicF3 (Fig. 7E). Cells producing wt DicF were highly filamentous and also had a “bloated” morphology (Fig. 7E), appearing greater in width (diameter) than control cells. Cells expressing DicF3 were also filamentous but appeared to be more uniform and similar in width to control cells (Fig. 7E). DicF3 does not regulate *xylR* or *pykA*, so, formally, loss of repression of these targets could account for differences in morphology between DicF- and DicF3-expressing cells. However, *xylR* and *pykA* mutants have no overt growth phenotypes in the rich LB medium used for this experiment (46); thus, it is unlikely that regulation of these targets accounts for the loss of viability and the more dramatic effects on morphology in DicF-producing compared to DicF3-producing cells. We hypothesize that the mutation in DicF3 also relieves regulation of other gene products that impact cell shape or cell wall structure and that this accounts for the observed differences.

### Physiological effects of DicF and DicB.

**FIG 8:**
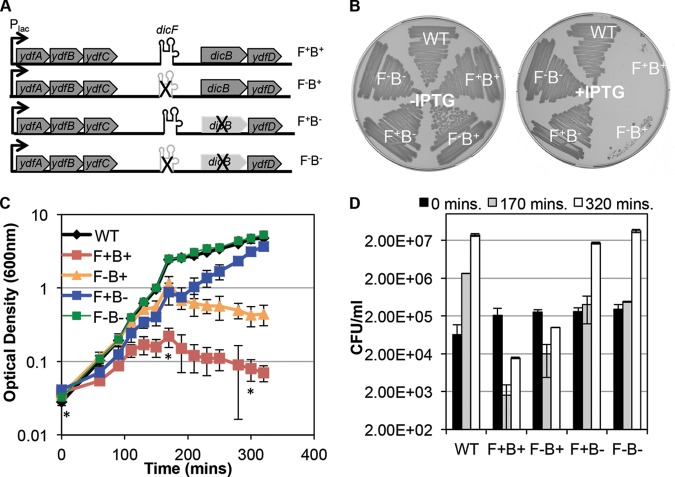
The *dicBF* chromosomal locus and roles of DicF and DicB in cell physiology. (A) Cartoon illustrating the chromosomal arrangement of the *dicBF* locus with the specific deletions depicted. A P_lac_ promoter was used to replace the native *dicBF* promoter (F^+^B^+^) in an otherwise wt strain. Gene *dicF* or gene *dicB* was deleted in this context to create an F^−^B^+^ strain or an F^+^B^−^ strain, respectively. Both genes were mutated in this context to make an F^−^B^−^ strain. (B) The wild-type strain (WT) and the four constructs described for panel A were streaked on LB plates with or without 0.5 mM IPTG. (C) The strains described above were grown in liquid LB medium, 0.5 mM IPTG was added at time 0, and growth was monitored over time. (D) The viability of the strains described for panel C was measured at the time points indicated by asterisks.

DicF and DicB are produced from the same operon, and both have been reported to inhibit cell division (14). To further explore the physiological functions of these products, we examined growth phenotypes of cells expressing *dicF* and/or *dicB* in the context of the intact operon. Since no signal that induces expression of the *dicBF* operon has been identified (it is not induced by SOS-inducing compounds [25]) (P. T. Ragunathan and C. K. Vanderpool, unpublished data), we inserted an inducible, P_lac_ promoter upstream of *ydfA* (Fig. 8A), thus replacing the promoter that is repressed by DicA and DicC (47, 48). We made constructs with a deletion of *dicF* or of *dicB* or of both genes (as previously described [49]), leaving only an 82-nt “FRT scar” sequence at each deletion site (Fig. 8A). We then assayed the growth of these strains on LB agar and in LB liquid medium upon induction of the operon. Induction of the wt operon (*dicF* positive [F^+^] and *dicB*^+^ [F^+^B^+^]; Fig. 8A) was extremely toxic, and no growth was observed on plates (Fig. 8B; +IPTG). Deletion of *dicB* (F^+^B^−^) in the context of this inducible operon largely relieved growth inhibition, whereas deletion of *dicF* (F^−^B^+^) allowed only very slight growth (Fig. 8B). Finally, deletion of both *dicF* and *dicB* (F^−^B^−^) relieved the growth inhibition, indicating that *dicF* and *dicB* are primarily responsible for the toxicity conferred by this operon. Using liquid medium, we observed that even in the absence of the inducer, the F^+^B^+^ strain was slightly growth inhibited (see Fig. S5B in the supplemental material). When strains were grown in LB liquid medium and exposed to inducer in early log phase, the F^+^B^+^ strain was severely growth inhibited (Fig. 8C) and showed 100-fold-reduced viability after ~2.5 h of induction (Fig. 8D). The F^+^B^+^ strain also showed the extensively filamented and bloated morphology (see Fig. S5C) observed in DicF-overproducing strains (Fig. 7E). The induced F^−^B^+^ strain was still growth inhibited but not as severely growth inhibited as the F^+^B^+^ strain (Fig. 8B). The F^−^B^+^ cells showed an ~10-fold reduction in viability (Fig. 8D) and displayed extensive filamentation (see Fig. S5C). Growth of the F^+^B^−^ cells was more inhibited than that of the control (wild-type [WT] and F^−^B^−^; Fig. 8C) strains, but the cells were less growth impaired than cells of either the F^+^B^+^ strain or the F^−^B^+^ strain (Fig. 8C). Moreover, F^+^B^−^ cells did not exhibit a decrease in CFU counts per milliliter at 2.5 h postinduction and in fact had grown to nearly wt levels by the end of the experiment (Fig. 8D; compare WT and F^+^B^−^ results). Consistent with this observation, the F^+^B^−^ cells were not filamentous (see Fig. S5C). In contrast with the DicF-associated filamentation observed when DicF was ectopically expressed on its own (Fig. 7), these results suggest that, expressed in the context of the intact operon, the presence of DicF by itself (i.e., without DicB) is not sufficient to strongly affect cell division. Instead, when the entire operon is induced, the cell division defect and most of the toxic effects appear to be due to the presence of DicB. Using Northern blots, we assessed levels of DicF in wt, F^+^B^+^, F^−^B^+^, F^+^B^−^, and F^−^B^−^ strains as well as in a strain expressing DicF from the P_lac_-*dicF* plasmid. While DicF was readily detected in cells carrying the P_lac_-*dicF* plasmid, it was not detected during IPTG-mediated induction of the chromosomal constructs shown in Fig. 8A (data not shown). This strongly suggests that the constructs illustrated in Fig. 8A do not produce DicF at levels sufficient to reproduce the DicF-associated phenotypes shown in Fig. 7. The F^−^B^−^ cells were not growth inhibited (Fig. 8C), and both CFU counts per milliliter (Fig. 8D) and morphology (Fig. S5C) resembled those seen with the wt strain.

10.1128/mSystems.00021-15.5 Figure S5  Growth of strains containing P*_lac_*-*dicBF*. (A) Cartoon illustrating the chromosomal arrangement of the *dicBF* locus with the specific deletions depicted. (B) The growth of the strains in LB medium without IPTG. (C) Phase-contrast images of strains shown in Fig. S4B imaged before and 60 min after the addition of 0.5 mM IPTG. Download Figure S5, PDF file, 1.9 MB. Copyright © 2016 Balasubramanian et al. 2016 Balasubramanian et al. This content is distributed under the terms of the Creative Commons Attribution 4.0 International license.

### The *dicF* gene and the *dicBF* operon are present in many *E. coli* strains.

**FIG 9:**
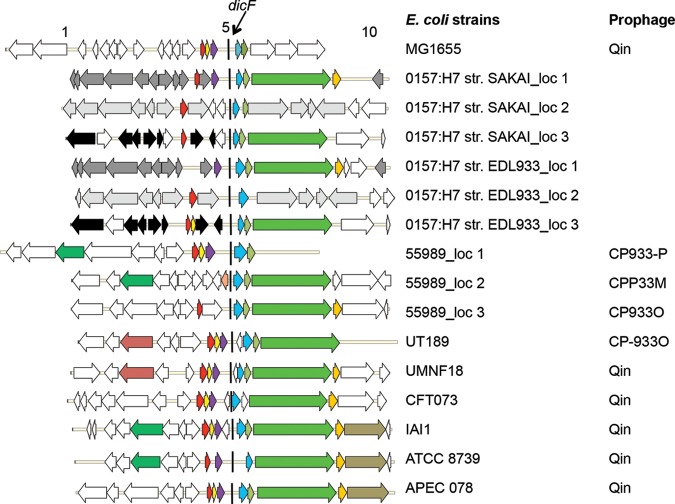
The *dicBF* locus is conserved in *E. coli* species in different prophages. The *dicF* sequences that were >99% identical among different *E. coli* species were obtained from NCBI. Nucleotide sequences 5 kb up- and downstream of *dicF* were then collected and aligned using Progressive Mauve. Open reading frames with sequence identity of >60% (verified by BLASTn) are indicated using the same colors in all prophages. The *dicBF* operon in MG1655 is arranged as follows: *ydfA* (in red), *ydfB* (in yellow), *ydfC* (in purple), *dicF* (indicated by a black line), *dicB* (in blue), and *ydfD* (in dark green). Genes that were not conserved are shown in white.

Since ectopic expression of DicF or the *dicBF* operon is toxic to *E. coli* cells, we wondered whether this sRNA or this operon would be conserved in other *E. coli* strains. The genetic region encompassing *dicBF* is similar to the bacteriophage P22 *immC* locus (48). Sequences that hybridize to probes from the *dicF* region were previously identified in several *E. coli* and *Shigella* species, but these sequences were not fully characterized (12). More recently, conservation of sRNAs, including DicF, was examined for 27 *E. coli* and *Shigella* strains (50). In this study, DicF was identified as a conserved sRNA in 17 of the 27 *E. coli* and *Shigella* genomes analyzed. We chose 10 different *E. coli* strains with DicF-like sRNAs for further analysis. Sequences 5 kb upstream and downstream of the predicted 5′ end of *dicF* were obtained from these 10 genomes and aligned using Progressive Mauve (51), and genes neighboring *dicF* were examined (Fig. 9). First, we noted that *dicF* and parts of the *dicBF* operon were present in multiple contexts within different prophages (Fig. 9). Second, *E. coli* strains carrying prophages closely resembling Qin (defined by NCBI and BioCyc annotations) from *E. coli* K-12 (UMNF18, CFT073, IAI1, ATCC 8739, and APEC 078) possessed the same *dicF* flanking genes, namely, *ydfA*, *ydfB*, and *ydfC* upstream and *dicB* and *ydfD* downstream (except the ATCC 8739 strain, which lacked *ydfB*). Third, some pathogenic *E. coli* strains (the O157:H7 Sakai and O157:H7 EDL933 strains) contained multiple copies of *dicF* and parts of the *dicBF* locus in different prophages (Fig. 9). Thus, it appears that despite their toxicity, *dicF* and the *dicBF* operon are widely retained in resident prophages in many *E. coli* strains and that some strains in fact have multiple copies of this locus.

## DISCUSSION

Despite being one of the earliest sRNAs identified in bacteria, DicF has remained poorly characterized for more than two decades. In this study, we directly tested and validated the hypothesis that DicF regulates *ftsZ* translation by an antisense base pairing mechanism. Moreover, we define two additional targets of DicF, *xylR* and *pykA* mRNAs, and show that different regions of DicF are responsible for regulating different targets. The 3′ end of DicF base pairs with *ftsZ* and *pykA* mRNAs, while the 5′ end is required for pairing with *xylR* mRNA. Analyses of viability and morphology of cells expressing DicF revealed that wt DicF is toxic, causing both filamentation and loss of viability (Fig. 7). Using mutant *dicF* alleles, we demonstrated that repression of *ftsZ* mRNA is responsible for filamentation but that the bloated cell morphology and loss of viability must be due to regulation of other unidentified targets (Fig. 7). When the entire operon containing *dicF* was ectopically expressed, toxicity that was even more dramatic than that seen with expression of *dicF* alone (Fig. 7) was observed (Fig. 8). Cell viability was reduced by more than 2 orders of magnitude when the entire *dicBF* operon was expressed, and this effect was due in large part to *dicB* (Fig. 8D). Interestingly, we identified DicF and DicB homologs in numerous *E. coli* chromosomes, sometimes in multiple independent prophage-encoded loci (Fig. 9). In contrast, BLAST searches of bacteriophage genome databases (NCBI and EMBL, containing ~2,000 sequenced phage genomes) failed to identify hits with significant similarity to DicB or DicF (data not shown). These observations hint that whatever function the *dicBF* operon served in the ancestral bacteriophage was less useful than the function it serves in extant *E. coli* hosts, where it is conserved in the context of defective prophages.

It is interesting to contemplate how sRNAs like DicF, which were brought into bacterial chromosomes on mobile genetic elements, have evolved to regulate bacterial genes, including some in the “core genome” (the set of genes shared by all strains of a given species). The InvR sRNA is carried on *Salmonella* pathogenicity island 1 (SPI1), and its expression is controlled by the major SPI1-encoded virulence regulator HilD (52). InvR, in turn, regulates the *Salmonella ompD* gene, encoding the abundant outer membrane porin OmpD (52). Another example is IpeX sRNA, which is carried on the *E. coli* cryptic prophage DLP12 (53). IpeX represses translation of *E. coli* *ompC* and *ompF* mRNAs, coding for the two major *E. coli* porins (53). Like DicF, both InvR and IpeX require Hfq for the base pairing-dependent regulation of at least some of their target mRNAs (52, 53). These observations suggest that horizontally acquired regulatory RNAs have evolved to use host cofactors (e.g., Hfq and RNase E) in order to regulate genes on the host chromosome. Maintenance of these regulatory interactions through evolutionary conservation likely reflects that the regulation improves the fitness of the host under some conditions. Understanding those conditions and the fitness benefits conferred is an ongoing challenge.

The considerations mentioned above become even more complex when we consider that the *dicBF* locus is present in multiple copies in several *E. coli* strains (Fig. 9). In addition to being present in Qin-like prophages (as in *E. coli* K-12), the *dicBF* locus is found in prophage islands dubbed “CP-933,” for “cryptic prophage 933” (21, 54, 55). The CP-933O, CP-933M, and CP-933P islands that carry *dicBF* are lambdoid prophages that are ~80 kb, 45 kb, and 57 kb in length, respectively (21, 55). Interestingly, the CP-933O prophage is thought to be a fusion of at least 2 prophages, one of which contains Qin-like genes (54, 55). Although these prophages are defective, their genomes are twice the size of Qin and are as large as or bigger than the genome of the ancestral lambda-like phage. A study analyzing the prophages of *E. coli* O157:H7 revealed that some defective prophages actually retain the ability to be excised and can be transferred to other bacteria (56). Thus, although these prophages may contain some mutations or losses that prevent their full functionality as lambdoid phage, some clearly retain functions that could facilitate spread of their genes to other strains. It would be fascinating to study how newly horizontally acquired sRNA regulators integrate into the host’s existing regulatory circuitry.

We observed that, expressed ectopically, DicF and DicB are independently toxic to *E. coli*. Expressed together by induction of the *dicBF* operon from a heterologous promoter, their effects are compounded and reduce viability of cells by more than 100-fold. Our studies showed that wt DicF caused filamentation and a morphological defect suggestive of problems with the cell wall (Fig. 7E). Cells expressing a DicF mutant that still regulated *ftsZ* were filamentous but otherwise had normal morphology and continued to increase in biomass (Fig. 7). Our interpretation of these results is that DicF targets other as-yet-unidentified genes involved in cell shape or chromosome segregation, since mutations in such genes have been shown to yield an elongated and bloated morphology (57) similar to that of cells expressing DicF. The only reported function of DicB is inhibition of FtsZ ring assembly through interactions with MinC (58–61) and ZipA (62). We found that ectopic expression of DicB is very toxic (Fig. 8). Whether this toxicity is due simply to inhibition of cell division, which eventually causes lysis, or to other unknown functions of DicB remains to be discovered.

Toxin-antitoxin (TA) systems represent another notable example of toxic prophage-encoded functions that are evolutionarily conserved on bacterial chromosomes (63). The toxins of TA systems become active only under certain conditions, where they inhibit growth and can contribute to a dormant state that is associated with bacterial persistence (64). Like TA systems, the *dicBF* operon is not active under standard laboratory growth conditions, owing to repression by DicA (48). We speculate that, similarly to TA systems, the *dicBF* operon is expressed under very specific environmental conditions and that the activities of the sRNA DicF and small protein DicB are beneficial to the host (on either the single-cell level or population level) under those conditions. Wang et al. (25) published a study consistent with this hypothesis in which they showed that the cryptic prophages in *E. coli* enhanced resistance to a variety of environmental stresses. They reported that Qin prophage and, specifically, DicB increased the resistance of *E. coli* to certain β-lactam antibiotics (25). Though we could not reproduce this particular result (data not shown), perhaps due to our use of a different strain background, it remains our hypothesis that the *dicBF* locus is conserved in numerous *E. coli* strains because it confers a fitness advantage under some conditions.

Finally, it is worth noting that limitations of widely used experimental and computational techniques inhibit faster progress in defining functions for bacterial sRNAs. In this study, we took a combined approach, using both RNA-Seq and biocomputational algorithms to identify DicF targets. Of 17 target candidates that we selected for further validation (based on experimental or computational predictions), we found only 2 new mRNAs that are directly regulated by base pairing with DicF (using a criterion of ≥2-fold regulation). Genetic and phenotypic analyses suggest that there are additional DicF targets that play important roles in the physiological effect of DicF. Transcriptomic approaches, including use of microarrays (65) or RNA-Seq (2, 66, 67), are certainly powerful approaches for defining sRNA target candidates, but they miss targets expressed at low basal levels or whose mRNA stability is not substantially changed by interactions with the sRNA. Moreover, there are often abundant indirect effects on gene expression from even short-term ectopic expression of sRNAs. Computational algorithms can in some cases accurately predict mRNA targets for sRNAs. Indeed, *xylR* was accurately predicted as a DicF target by computational prediction but did not appear in the RNA-Seq data because it was not highly expressed under our experimental conditions. However, this example represents an exception, since other computationally predicted targets were not posttranscriptionally regulated by DicF (Table 1 and Fig. 1). These results highlight the high rates of false negatives and false positives produced even using a combination of global approaches for sRNA target identification, and this has been typical of our experience in characterization of several sRNAs in *E. coli* (reference 68 and unpublished results). Development of new tools for the more rapid and accurate characterization of sRNA target regulons would greatly facilitate efforts to define functions for hundreds of fascinating bacterial sRNAs.

## MATERIALS AND METHODS

### Strain and plasmid construction.

All strains and plasmids used in this study are summarized in Table S3 in the supplemental material, and the oligonucleotides (obtained from IDT) used in this study are listed in Table S4. The strains used in this study are derivatives of the Δ*lac* DJ480 strain (D. Jin, National Cancer Institute), which was derived from MG1655. All lambda red recombination methods were performed as described in reference 69.

10.1128/mSystems.00021-15.8 Table S3  Strains and plasmids used in this study. Download Table S3, DOC file, 0.1 MB. Copyright © 2016 Balasubramanian et al. 2016 Balasubramanian et al. This content is distributed under the terms of the Creative Commons Attribution 4.0 International license.

10.1128/mSystems.00021-15.9 Table S4  Oligonucleotides used to make strains and plasmids. Download Table S4, DOCX file, 0.02 MB. Copyright © 2016 Balasubramanian et al. 2016 Balasubramanian et al. This content is distributed under the terms of the Creative Commons Attribution 4.0 International license.

The predicted targets of DicF were recombined into PM1205 as described in reference 32. Briefly, sequences of targets spanning the +1 site to 10 or 20 amino acids of coding sequence were amplified with oligonucleotides with homology to the P_BAD_ and *lacZ* sequences. Lambda red recombineering was used to generate the fusions described for Fig. 1. Sequences of the *carB* (O-DB429/430), *rlmN* (O-DB433/439), *pgaA* (O-DB427/428), *ppk* (O-DB425/426), *psiE* (O-DB391/392), *clcA* (O-DB393/394), *glmS* (O-DB441/442), *cyoA* (O-DB445/446), *pykA* (O-DB475/476), *rbsD* (PR108/109), *rbsA* (O-DB414-a/415), *ptsP* (O-DB447/448), *glpK* (O-DB418/419), *xylR* (O-DB385/386), and *pfkA* (O-DB360/362) genes were PCR amplified using oligonucleotides (names shown in parentheses) and were integrated into PM1205 to create DB196, DB199, DB197, DB198, DB191, DB192, DB193, DB215, DB214, DB228, DB219, DB195, DB190, DB194, JH193, JH256, DB189, and DB177.

The *qin*::*kan* deletion in Fig. 2 was created by lambda red recombination using primers O-DB413/O-DB414. This mutation was then moved into DB189 (*xylR*′*-*′*lacZ*), DB228 (*pykA*′*-*′*lacZ*), and DB229 (*ftsZ*′*-*′*lacZ*) via P1 transduction to create DB120, DB237, and PR124, respectively. The *ftsZ*′*-*′*lacZ* fusion containing only 4 amino acids (DB229) and *ftsZ_comp23_*′*-*′*lacZ* (PR130) were created in PM1205 as described above using oligonucleotides O-DB503/O-DB504 and O-PR151/O-PR152, respectively. The *xylR_3TRUNC_*′*-*′*lacZ* (DB202) fusion contained the +1 site to the first amino acid of *xylR* and was created with oligonucleotides O-DB385 and O-DB436. The *xylR_comp11_*′*-*′*lacZ* fusion (DB227) was constructed in PM1205 with primers O-DB385 and O-DB458 (containing the *xylR11* mutation). The *rne131* mutant obtained from the Masse laboratory (EM1377 [33]) was linked to a kanamycin resistance cassette (inserted in the intergenic region between *rne131* and the downstream *flgL* gene) by Maksym Bobrovskyy in our laboratory to yield strain MB10 (68). This mutation was then moved into DB189, DB228, and DB229 to yield DB207, DB239, and PR125, respectively. Similarly, the Δ*hfq*::*cat* mutation (37) was moved by P1 transduction into DB189, DB228, and DB229 to yield DB206, DB238, and PR127, respectively.

The Δ*xylR* strain was obtained from the Keio collection (46). The DB176 strain was created by recombineering of a *dicF*::*kan* PCR product amplified using O-DB358/O-DB359. The *pykA*_comp23′*-*′_*_lacZ_* fusion was made as described above using oligonucleotides O-DB475/O-DB526. A P_lac_ promoter sequence linked to a chloramphenicol resistance cassette (70) was amplified using oligonucleotides O-DB479 and O-DB480 and recombined into a *lacI*^q*+*^ strain to make DB240. PCR products generated using oligonucleotides O-DB508/O-DB509 (to make a Δ*dicB*::*kan* deletion) and O-DB521/O-DB522 (to make a Δ*dicF*::*kan* deletion) were recombined independently into DB240 to create DB241 and DB252. Further, the kanamycin cassettes in DB241 and DB252 were removed using pCP20 (49), generating strains DB243 (Δ*dicB*) and DB247 (Δ*dicF*), respectively. Lastly, the *dicF*::*kan* PCR product was once again recombined into DB243 and the kanamycin cassette removed to create the double Δ*dicF* Δ*dicB* mutant (DB248).

The P_lac_-vector and the P_lac_-*dicF* plasmids were obtained from the Gottesman laboratory (45). P_lac_*-dicF* mutant alleles used in this study were generated using a QuikChange mutagenesis II kit (Stratagene). The oligonucleotides used to create the individual mutants are described in Table S4.

The Cp19-*xylR* strain was made by amplifying the kanamycin-linked Cp19 promoter from JNB034 (33) using oligonucleotides O-DB463 and O-DB464 and by recombineering of the linear PCR product in place of the *xylR* promoter. Cp19-*xylR* was moved to a *lacI*^q*+*^ strain to create DB223. The Cp19-*pykA lacI*^q^ strain (DB224) was created in a similar manner using oligonucleotides O-DB459 and O-DB460. Strains DB223 and DB224 were used in experiments represented in Fig. S1A and B in the supplemental material, respectively.

### Computational predictions of DicF targets.

The four sRNA target prediction programs, TargetRNA (26), IntaRNA (27), sTarPicker (29), and CopraRNA (28), were used to generate lists of potential DicF targets. The search window interrogated for potential interactions with DicF was set at 100 nucleotides (nt) upstream of the start codon to 20 amino acids into the coding sequence. Genes with interactions that were predicted by at least two programs with a *P* value of ≤0.05 were chosen for further analyses.

### RNA-Seq experiments and data analyses.

A Δ*dicF lacI*^q^ strain harboring the vector control or the plasmid containing *dicF* was grown to an optical density at 600 nm (OD_600_) of ~0.1 in LB with ampicillin. Three biological replicates were performed. IPTG (0.5 mM) was added to the cultures to induce DicF production. RNA was harvested 20 min after induction, treated with DNase (Ambion), and checked for integrity on a 1% agarose gel. Library construction and sequencing were performed at the W. M. Keck Center for Comparative and Functional Genomics at the University of Illinois at Urbana—Champaign. Ribosomal RNA was removed from 1 µg of total RNA using a Ribozero rRNA Removal Meta-Bacteria kit (Epicentre Biotechnologies), and the mRNA-enriched fraction was converted to indexed RNA-Seq libraries with a ScriptSeq v2 RNA-Seq library preparation kit (EPICENTRE Biotechnologies). The libraries were pooled in equimolar concentrations and were quantitated by quantitative PCR (qPCR) using a library quantification kit (Illumina compatible; Kapa Biosystems) and sequenced for 101 cycles plus 7 cycles for the index read using a HiSeq 2000 sequencing system and TruSeq SBS version 3 reagents. The Fastq files were generated with Casava 1.8.2 (Illumina). The computational program Rockhopper (31) was used to analyze the RNA-Seq data. Details of normalization procedures can be found in the publication describing Rockhopper (31). The cutoffs of >100 normalized sequence reads and ≥2-fold differential expression were chosen based on our experience with other sRNAs. We have found that results from candidate targets showing very low normalized read counts or low fold changes between control and experimental samples in RNA-Seq experiments are more likely to be false positives. RNA-Seq data were submitted to the Gene Expression Omnibus (GEO) at the National Center for Biotechnology Information (NCBI).

### β-Galactosidase assays.

Strains were grown overnight in Terrific broth (TB) medium (with 100 µg/ml ampicillin for plasmid selection) and were subcultured into fresh media with antibiotics and grown to mid-logarithmic phase. When cultures reached an OD_600_ of ~0.3, IPTG (isopropyl-β-d-thiogalactopyranoside; Sigma Aldrich) was added at a final concentration of 0.1 mM. Samples were harvested after 1 h and assayed for β-galactosidase activity as described previously (71). All experiments were conducted in triplicate.

### RNA extraction and Northern blot analysis.

Strains carrying plasmids were grown overnight in LB with ampicillin. They were then subcultured into fresh media with antibiotics and grown to mid-log phase. When cultures reached an OD_600_ of ~0.3, 0.5 mM IPTG was added, and samples were harvested at different time points. RNA was extracted by the hot phenol method as described in reference 72.

Northern blot analysis was carried out as described in reference 73. Briefly, 7 µg total RNA (for DicF) or 10 µg total RNA (for *xylR* and *pykA* mRNAs) was run on acrylamide gels and 1% agarose gels using 1× Tris-acetate-EDTA (TAE) or 1× MOPS (morpholinepropanesulfonic acid) buffer, respectively. RNA in acrylamide gels was transferred to a 0.2-µm-pore-size membrane (Whatman) in 0.5× TAE buffer by electrophoresis. RNA in agarose gels was transferred by capillary transfer using 20× SSC (1× SSC is 0.15 M NaCl plus 0.015 M sodium citrate). Following transfer, the membranes were probed overnight with biotinylated DNA oligonucleotides (IDT) complementary to the respective RNAs. Detection was carried out according to the instructions for the Brightstar Biodetect kit (Ambion).

### LB growth inhibition.

Strains carrying plasmids with wt *dicF*, *dicF* mutants, or chromosomal P*_lac_*-*dicBF* constructs were grown to an OD_600_ of ~0.1. IPTG (0.5 mM) was used to induce expression of DicF or the *dicBF* operon. Growth was monitored over time by measuring the OD_600_ of cultures until they reached stationary phase.

### Xylose, glucose, and fructose growth inhibition.

Δ*dicF lacI*^q^ strains harboring the vector or the *dicF*, *dicF3*, or *dicF9* plasmid were streaked on M63 xylose, glucose, and fructose medium with and without 0.5 mM IPTG. Plates were imaged after 22 h of incubation.

### Phase-contrast microscopy.

Cultures were grown to the indicated time points. Cell cultures (500 μl to 1 ml) were collected by centrifugation. The cell pellet was resuspended with 1× phosphate-buffered saline (PBS), washed, and resuspended in 1× PBS. The resuspended cells (1 µl) were pipetted onto a 24-by-50-mm no. 1.5 coverslip (Fisher Scientific; catalog no. 12-544E). A 1.5% agarose gel pad (in 1× PBS) was laid on the cells for immobilization. Cells were then imaged using an inverted epifluorescence microscope (Nikon Instruments Eclipse TE2000-E) and an electron microscopy charge-coupled-device (EMCCD) camera (Photometrics; Cascade 512). A 100× numerical aperture (NA) 1.40 oil immersion phase-contrast objective (Nikon Instruments Plan Apo 100×/1.40 Oil) was used in conjunction with a ×2.5 lens in front of the camera. The microscope and camera were controlled using Metamorph software (Molecular Devices). Each sample was imaged at multiple locations.

### Microarray data accession number.

RNA-Seq data were submitted to the Gene Expression Omnibus (GEO) at the National Center for Biotechnology Information (NCBI; http://www.ncbi.nlm.nih.gov/geo/); the GEO accession number is GSE76916.
