# Supplementary material for: A Prophage-Encoded Small RNA Controls Metabolism and Cell Division in Escherichia coli
Source: mSystems. 2016 Feb 9;1(1):e00021-15. doi: 10.1128/mSystems.00021-15 (PMC5069750; doi:10.1128/mSystems.00021-15)
Supplement: Table S1 [file sys001162003st6.docx]

|  | Computational programs and predicted interactions (DicF, mRNA)^a^ | | | |
| --- | --- | --- | --- | --- |
| **Gene name** | **Target RNA** | **IntaRNA** | **Copra RNA** | **Starpicker** |
| *rbsC* | (22 to 40, -10 to 4) | NP **^b^** | (38 to 44, -10 to -4) | NP |
| *manX* | NP | (36 to 53, 45 to 62) | (45 to 62, 35 to 52) | (35 to 53, 34 to 53) |
| *xylR* | (5 to 18, 7 to 20) | (6 to 15, 10 to 19) | NP | (5 to 18, 7 to 20) |
| *pykA* | NP | (37 to 49, -2 to -18) | (39 to 51, -4 to -17) | NP |
| *glpK* | NP | (6 to 52, -158 to -192) | (25 to 61, -158 to -192) | NP |
| *nuoL* | NP | NP | (11 to 21, -33 to -43) | (18 to 53, -33 to -94) |
| *ptsP* | (22 to 34, -64 to -72) | NP | NP | (10 to 22, -18 to -34) |
| *ftsZ* | NP | (6 to 52, 9 to 63) | (26 to 62, 7 to 53) | NP |
| *ppK* | (21 to 37, -1 to -17) | NP | (38 to 46, -8 to -16) | NP |
| *rlmN* | NP | (2 to 15, 27 to 41) | (10 to 24, 27 to 42) | (1 to 16, 26 to 42) |
| *mtr* | (7 to 22, 15 to 30) | (6 to 15, 23 to 32) | NP | NP |
| *carB* | (3 to 44, 17 to -22) | (3 to 47, -25 to 17) | (51 to 64, -27 to -43) | (2 to 49, -27 to 18) |
| *pgaA* | (35 to 39, -3 to -17) | NP | (38 to 47, -6 to -15) | NP |
| *psiE* | (5 to 19, -4 to 12) | NP | NP | (7 to 27, -8 to 3) |

**Table S1**. **Predicted interactions between DicF and targets.** Regions of base pairing interactions between selected target mRNAs and DicF.

**^a^** The numbers listed are base pairs on DicF (relative to the +1 site) involved in the interactions followed by nucleotides on the target (relative to the first nucleotide of the start codon)

**^b^** NP: Not predicted by the program
