# Supplementary figures and images for: A Prophage-Encoded Small RNA Controls Metabolism and Cell Division in Escherichia coli
Source: mSystems. 2016 Feb 9;1(1):e00021-15. doi: 10.1128/mSystems.00021-15 (PMC5069750; doi:10.1128/mSystems.00021-15)

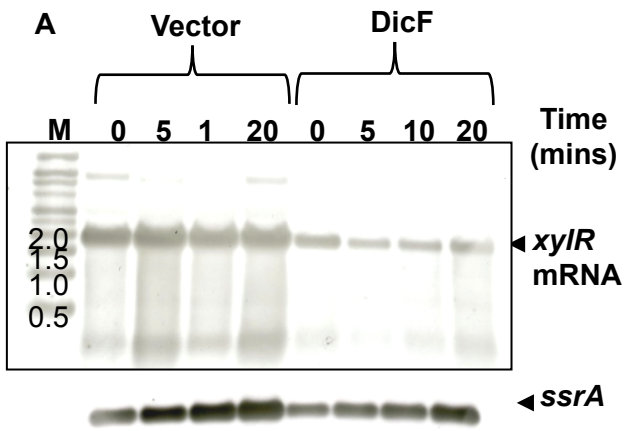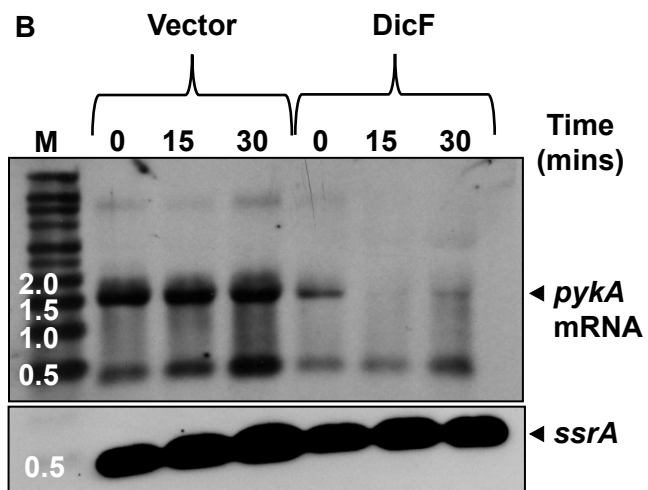

Supplement: Figure S1 [file sys001162003sf1.pdf]

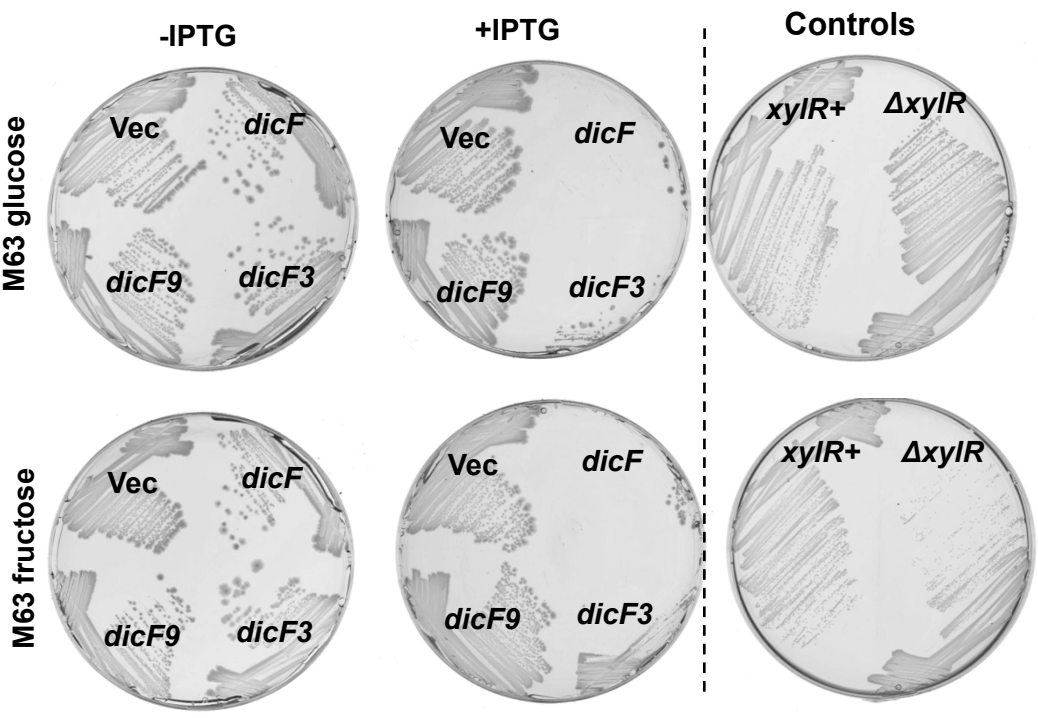

Supplement: Figure S2 [file sys001162003sf2.pdf]

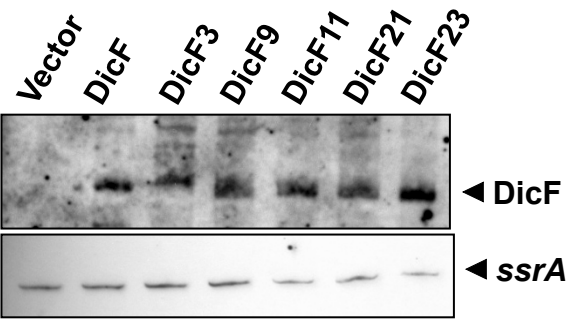

Supplement: Figure S3 [file sys001162003sf3.pdf]

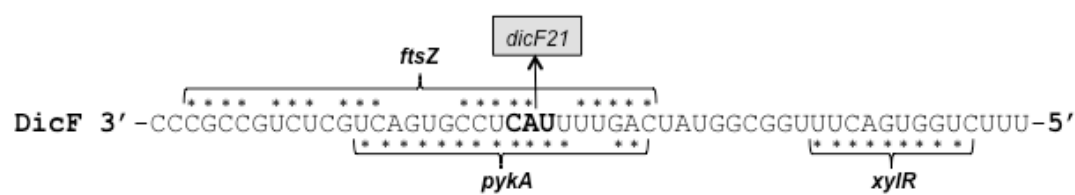

Supplement: Figure S4 [file sys001162003sf4.pdf]

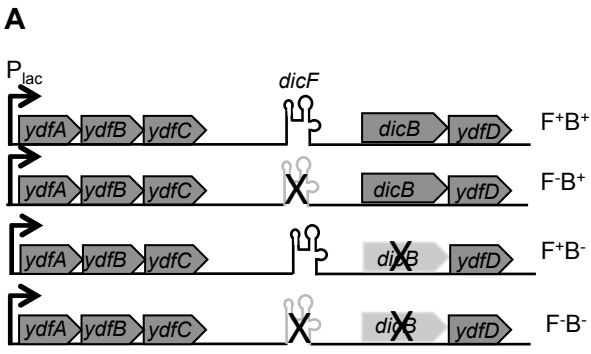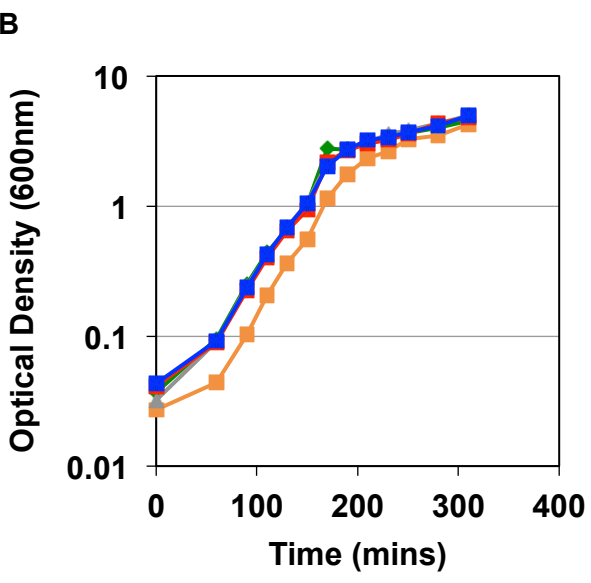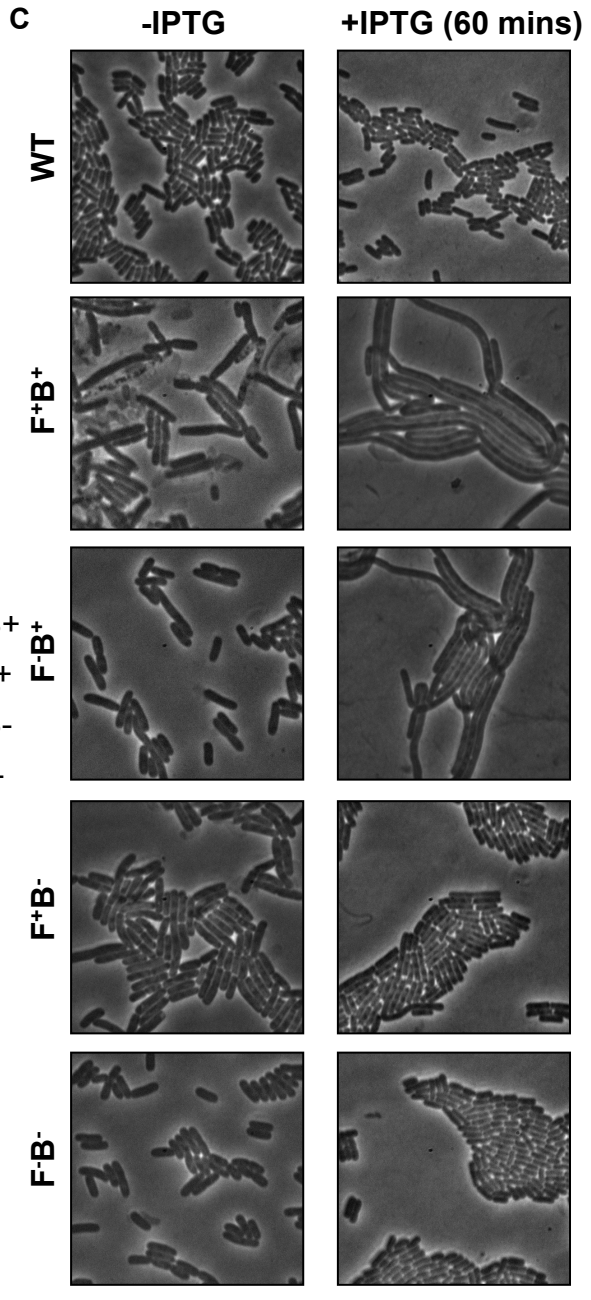

Supplement: Figure S5 [file sys001162003sf5.pdf]
