# Supplementary material for: A Prophage-Encoded Small RNA Controls Metabolism and Cell Division in Escherichia coli
Source: mSystems. 2016 Feb 9;1(1):e00021-15. doi: 10.1128/mSystems.00021-15 (PMC5069750; doi:10.1128/mSystems.00021-15)
Supplement: Table S3 [file sys001162003st8.doc]

**Supporting Material**

Table S3. Strains and plasmids used in this study.

| **Strain** | **Description** | **Source/Reference** |
| --- | --- | --- |
| DJ480 | MG1655 Δ*lac* X74 | D. Jin, NCI |
| NM200 | DJ480, mini λ::Cm (carries λ red recombination functions) | N. Majdalani, NCI |
| NM2000 | DJ480, mini λ::Cm, mal::lacI^q^ | N. Majdalani, NCI |
| PM1205 | DJ480 P_BAD_::*cat-sacB*::*lacZ*, mini λTet | [1] |
| DB120 | DB189 Δ*qin::kan* | This study |
| DB176 | DJ480 *ΔdicF, lacI^q^* | This study |
| DB177 | PM1205 *pfkA′-′lacZ* | This study |
| DB189 | PM1205 *xylR′-′lacZ* | This study |
| DB190 | PM1205 *ptsP′-′lacZ* | This study |
| DB192 | PM1205 *psiE′-′lacZ* | This study |
| DB193 | PM1205 *clcA′-′lacZ* | This study |
| DB194 | PM1205 *glpK′-′lacZ* | This study |
| DB195 | PM1205 *rbsA′-′lacZ* | This study |
| DB196 | PM1205 *carB′-′lacZ* | This study |
| DB197 | PM1205 *pgaA′-′lacZ* | This study |
| DB198 | PM1205 *ppk′-′lacZ* | This study |
| DB199 | PM1205 *rlmN′-′lacZ* | This study |
| DB202 | PM1205 *xylR3trunc′-′lacZ* | This study |
| DB206 | DB189 Δ*hfq::cat* | This study |
| DB207 | DB189 *rne131::kan* | This study |
| DB214 | PM1205 *cyoA′-′lacZ* | This study |
| DB215 | PM1205 *glmS′-′lacZ* | This study |
| DB219 | PM1205 *rbsD′-′lacZ* | This study |
| DB223 | DB221 *Cp19-xylR* λattB::*lacI^q^* | This study |
| DB224 | DB221 *Cp19-pykA* λattB::*lacI^q^* | This study |
| DB227 | PM1205 *xylR_comp11_′-′lacZ* | This study |
| DB228 | PM1205 *pykA′-′lacZ* | This study |
| DB229 | PM1205 *ftsZ′-′lacZ* | This study |
| DB237 | DB228 Δ*qin::kan* | This study |
| DB238 | DB228 Δ*hfq::cat* | This study |
| DB239 | DB228 *rne131::kan* | This study |
| DB240 | NM2000 *cat-P_lac_-dicBF* | This study |
| DB241 | DB240 Δ*dicB::kan* | This study |
| DB243 | DB240 Δ*dicB* | This study |
| DB247 | DB240 Δ*dicF* | This study |
| DB248 | DB240 Δ*dicB* Δ*dicF* | This study |
| DB252 | DB240 Δ*dicF::kan* | This study |
| DB255 | PM1205 *pykA_comp23′-′lacZ_* | This study |
| JH193 | *Cp19-manX′-′lacZ* | [2] |
| JH256 | *Cp19-manXY′-′lacZ* | [3] |
| PR130 | PM1205 *ftsZ_comp23_′-′lacZ* | This study |
| PR124 | DB229 Δ*qin::kan* | This study |
| PR125 | DB229 Δ*rne131::kan* | This study |
| PR127 | DB229 Δ*hfq::cat* | This study |

| **Plasmids** | **Primers** | **Reference** |
| --- | --- | --- |
| P_lac_-vector | N/A | Gottesman laboratory, NIH |
| P_lac_-*dicF* | N/A | Gottesman laboratory, NIH |
| P_lac_-*dicF3* | O-DB349  O-DB350 | This study |
| P_lac_-*dicF* *8* | O-DB397  O-DB398 | This study |
| P_lac_-*dicF9* | O-DB408  O-DB409 | This study |
| P_lac_-*dicF11* | O-DB451  O-DB452 | This study |
| P_lac_-*dicF14* | O-DB487  O-DB488 | This study |
| P_lac_-*dicF15* | O-DB489  O-DB490 | This study |
| P_lac_-*dicF16* | O-DB491  O-DB492 | This study |
| P_lac_-*dicF21* | O-DB501  O-DB502 | This study |
| P_lac_-*dicF23* | O-DB530  O-DB531 | This study |

Literature Cited

1. Mandin, P. and S. Gottesman, *A genetic approach for finding small RNAs regulators of genes of interest identifies RybC as regulating the DpiA/DpiB two-component system.* Mol Microbiol, 2009. **72**(3): p. 551-65.

2. Rice, J.B. and C.K. Vanderpool, *The small RNA SgrS controls sugar-phosphate accumulation by regulating multiple PTS genes.* Nucleic Acids Res, 2011. **39**(9): p. 3806-19.

3. Rice, J.B., D. Balasubramanian, and C.K. Vanderpool, *Small RNA binding-site multiplicity involved in translational regulation of a polycistronic mRNA.* Proc Natl Acad Sci U S A, 2012. **109**(40): p. E2691-8.
