# Supplementary material for: A Prophage-Encoded Small RNA Controls Metabolism and Cell Division in Escherichia coli
Source: mSystems. 2016 Feb 9;1(1):e00021-15. doi: 10.1128/mSystems.00021-15 (PMC5069750; doi:10.1128/mSystems.00021-15)
Supplement: Table S4 [file sys001162003st9.docx]

Table S4. Oligonucleotides used to make strains and plasmids

| Name | Sequence 5'-3' |
| --- | --- |
| O-DB429 | ACCTGACGCTTTTTATCGCAACTCTCTACTGTTTCTCCATGCAGTACCGTAAAACCGCTA |
| O-DB430 | TAACGCCAGGGTTTTCCCAGTCACGACGTTGTAAAACGACGATAACAATCGGGCCCG |
| O-DB433 | ACCTGACGCTTTTTATCGCAACTCTCTACTGTTTCTCCATGTACAATGCAGCGCCCCCGG |
| O-DB434 | TAACGCCAGGGTTTTCCCAGTCACGACGTTGTAAAACGACCTCCCGCATCTGCTGACGGT |
| O-DB427 | ACCTGACGCTTTTTATCGCAACTCTCTACTGTTTCTCCATGAGAAAAGGATTTTATATGGACACTCTGC |
| O-DB428 | TAACGCCAGGGTTTTCCCAGTCACGACGTTGTAAAACGACAGTAAGAAGTTTCAAAGCCCA TTTGG |
| O-DB425 | ACCTGACGCTTTTTATCGCAACTCTCTACTGTTTCTCCATGCTCGCCATAATATCCAGGC |
| O-DB426 | TAACGCCAGGGTTTTCCCAGTCACGACGTTGTAAAACGACGCGTTCATTGAACGATAACC |
| O-DB391 | ACCTGACGCTTTTTATCGCAACTCTCTACTGTTTCTCCATTCGCCACTGTGAAGGAGGTACTG |
| O-DB392 | TAACGCCAGGGTTTTCCCAGTGACGACGTTGTAAAACGACCTCCACGCGCGGACGAGAGA |
| O-DB393 | ACCTGACGCTTTTTATCGCAACTCTCTACTGTTTCTCCATAACGTGCGAGTAAAATGCCG |
| O-DB394 | TAACGCCAGGGTTTTCCCAGTGACGACGTTGTAAAACGACCGGTGTTTCTAAAGAGGGAGTAT |
| O-DB414A | ACCTGACGCTTTTTATCGCAACTCTCTACTGTTTCTCCATGCTGTGGGCAAGCTTAATCG |
| O-DB415 | TAACGCCAGGGTTTTCCCAGTCACGACGTTGTAAAACGACCTGCTCCATCAGCCACGCTTT |
| O-DB441 | ACCTGACGCTTTTTATCGCAACTCTCTACTGTTTCTCCATGTCACATGGGATGAGGAGATAACATAATCTCCC |
| O-DB442 | TAACGCCAGGGTTTTCCCAGTCACGACGTTGTAAAACGACTTGCGCGATCGCGCCAACAA |
| O-DB445 | ACCTGACGCTTTTTATCGCAACTCTCTACTGTTTCTCCATACACTTTAAACGCCACCAGATC |
| O-DB446 | TAACGCCAGGGTTTTCCCAGTCACGACGTTGTAAAACGACTAACAGCGCAGAATTACAGCC |
| O-DB475 | ACCTGACGCTTTTTATCGCAACTCTCTACTGTTTCTCCATCTTATACGACATCCGAATGAGATTAATTTATCGC |
| O-DB476 | TAACGCCAGGGTTTTCCCAGTCACGACGTTGTAAAACGACAAGCCTTCTGGAcatGTAATACTCCG |
| O-DB447 | ACCTGACGCTTTTTATCGCAACTCTCTACTGTTTCTCCATGAAAGAGTTCGCGAGTGTGGTG  ATGTC |
| O-DB448 | TAACGCCAGGGTTTTCCCAGTCACGACGTTGTAAAACGAC  CTCATTCAGGCGTGGTGCGC |
| O-DB418 | ACCTGACGCTTTTTATCGCAACTCTCTACTGTTTCTCCATCTTTACCGGCGGcAGAGACAT  TCCTTAC |
| O-DB419 | TAACGCCAGGGTTTTCCCAGTCACGACGTTGTAAAACGACGACCGCGCGGGAGCTGGTGG |
| O-DB385 | ACCTGACGCTTTTTATCGCAACTCTCTACTGTTTCTCCATGATAAAAATTTTCTCAAAGCCGGT |
| O-DB386 | TAACGCCAGGGTTTTCCCAGTGACGACGTTGTAAAACGACTAATGTGATGCGGTGACGTT |
| O-DB360 | ACCTGACGCTTTTTATCGCAACTCTCTACTGTTTCTCCATgatttcattttgcattccaaag |
| O-DB362 | TAACGCCAGGGTTTTCCCAGTCACGACGTTGTAAAACGACGCTTGTCAACACACCGATTTTC |
| O-DB403 | ACCTGACGCTTTTTATCGCAACTCTCTACTGTTTCTCCATGCCGTTTATACCGGTGGGGC |
| O-DB377 | TAACGCCAGGGTTTTCCCAGTCACGACGTTGTAAAACGACGCCGCCGACGCCGATGACTT |
| O-DB504 | TAACGCCAGGGTTTTCCCAGTCACGACGTTGTAAAACGACTGGTTCAAACATAGTTTCTCTCCGATTTGTGCCTGTC |
| O-DB506 | TAACGCCAGGGTTTTCCCAGTCACGACGTTGTAAAACGACTGGTTCAAACATAGTTTCTCTCCGATTTCTGCCTGTC |
| O-DB503 | ACCTGACGCTTTTTATCGCAACTCTCTACTGTTTCTCCATAAGTGCGTATCGGCGCGCCG |
| O-DB436 | TAACGCCAGGGTTTTCCCAGTCACGACGTTGTAAAACGACTAATGTGATGCccaGACGTTTAGTAAAcat |
| O-DB458 | TAACGCCAGGGTTTTCCCAGTCACGACGTTGTAAAACGACTAATGTGATGCGCTGACGTTTAGTAAAcat |
| O-DB463 | GAGTATGATGGATGTACCGACCTTCTGGCAGTATATCGTTGTGTAGGCTGGAGCTGCTTCG |
| O-DB464 | ACCGGTAATACGTAACCGGCTTTGAGAAAATTTTTACAAATAACTATTATATCAACCCGGATCCCTCC |
| O-DB459 | GATTTACTTTGCGACGCGGTGCAAAATTCAGAGATAACTTGTGTAGGCTGGAGCTGCTTCG |
| O-DB460 | TGGCGATAAATTAATCTCATTCGGATGTCGTATAAGATTATAACTATTATATCAACCCGGATCCCTCC |
| O-DB358 | TTTCTGGTGACGTTTGGCGGTATCAGTTTTACTCCGTGACgtgtaggctggagctgcttc |
| O-DB359 | GCGCTCAGCCGCATTCACCACATCACAAAATTCACTTTAAATTCCGGGGATCCGTCGACC |
| O-DB479 | TCATCAATGAGTTATCTTTTACCACATCAATTATGTTAGCCCTGTGACGGAAGATCACTT |
| O-DB480 | CACCGTGCGGTGTGTTGATGCAAACAAGATTAGCCATGACGTGCTCAGTATCTTGTTATC |
| O-DB508 | atgAAAACGTTATTACCAAACGTTAATACGTCTGAAGGTTGTGTAGGCTGGAGCTGCTTC |
| O-DB509 | TcaTTGTGCACATCCTTTTGGCATCAGACGTAAACGAGCCAATTCCGGGGATCCGTCGACC |
| O-DB521 | CTTAAGTGACAACCCCGCTGCAACGCCCTCTGTTATCAATGTGTAGGCTGGAGCTGCTTC |
| O-DB522 | GTGCGCTCAGCCGCATTCACCACATCACAAAATTCACTTTATTCCGGGGATCCGTCGACC |
| O-DB349 | CAAGATACTGACGTCTTTCTCCAGACGTTTGGCGGTATCA |
| O-DB350 | TGATACCGCCAAACGTCTGGAGAAAGACGTCAGTATCTTG |
| O-DB397 | GGTATCAGTTTTACTCCGTctgTGCTCTGCCGCC |
| O-DB398 | CTTTAAAAAGGGCGGCAGAGCACAGACGGAGTAA |
| O-DB408 | GGTGACGTTTGGCGGTATCAGTTTTAgagCGTGACT |
| O-DB409 | AAAAAGGGCGGCAGAGCAGTCACGctcTAAAACTGATAC |
| O-DB451 | CAAGATACTGACGTCTTTCTGCTGACGTTTGGCGGTATCA |
| O-DB452 | TGATACCGCCAAACGTCAGCAGAAAGACGTCAGTATCTTG |
| O-DB487 | GGCGGTATCAGTTTTACTCGCTGACTGCTCTGCC |
| O-DB488 | GGGCGGCAGAGCAGTCAGCGAGTAAAACT |
| O-DB489 | GGCGGTATCAGTTTTACTCCGTCACTGCTCTGCC |
| O-DB490 | GGGCGGCAGAGCAGTGACGGAGTAAAACT |
| O-DB491 | CGGTATCAGTTTTACTCCGTGACTCGTCTGC |
| O-DB492 | GGGCGGCAGACGAGTCACGGAGTAAAACTGATA |
| O-DB501 | GTGACGTTTGGCGGTATCAGTTTATGTCCGTGACTG |
| O-DB502 | GGGCGGCAGAGCAGTCACGGACATAAACTGATACC |
| O-DB413 | CATGAAACACTAACGCTGCGCCGAGCGAATATAACTGGAAATCCATAA GTGTAGGCTGGAGCTGCTTC |
| O-DB414 | ATTTGGTGAAAGCAGCAGTATCGGGATGGCTGGGCACTGCGCTTGAAT ATTCCGGGGATCCGTCGACC |
| O-DB504 | TAACGCCAGGGTTTTCCCAGTCACGACGTTGTAAAACGACTGGTTCAAACATAGTTTCTCTCCGATTTGTGCCTGTC |
| O-DB506 | TAACGCCAGGGTTTTCCCAGTCACGACGTTGTAAAACGACTGGTTCAAACATAGTTTCTCTCCGATTTCTGCCTGTC |
| O-DB530 | GGCGGTATCAGTTTTACTCCCTGACTGCTCT |
| O-DB531 | GGGCGGCAGAGCAGTCAGGGAGTAAAACTGATA |
| O-DB527 | TAACGCCAGGGTTTTCCCAGTCACGACGTTGTAAAACGACAAGCCTTCTGGACATGTAATACTCCCTTGACTG |
| O-PR108 | ACCTGACGCTTTTTATCGCAACTCTCTACTGTTTCTCCATAGCGAAACGTTTCGCTGATGGAG |
| O-PR109 | TAACGCCAGGGTTTTCCCAGTCACGACGTTGTAAAACGAC ATGTCCCAGACGGGAGATCACC |
| O-PR151 | ACCTGACGCTTTTTATCGCAACTCTCTACTGTTTCTCCATAAGTGCGTATCGGCGCGCCGCTG |
| O-PR152 | TAACGCCAGGGTTTTCCCAGTCACGACGTTGTAAAACGACTGGTTCAAACATAGTTTCTCTCCCATTTGTGCCTGTCG |
| *dicF-bio* | /5BIO/AGATACTGACGTCTTTCTGGTCTGGTTTGGCGGTATCAGTTT |
| *xylR-bio* | /5bio/ggtaactttctgcaaggtgatacg |
| *pykA-bio* | /5BIO/GCGATCTGTTGCTGGGCCTAACGTGGTAACGATTTTTGTT |
| *ssrA*-bio | /5Bio/CGCCACTAACAAACTAGCCTGA |
